# BADGER: biologically-aware interpretable differential gene expression ranking model

**DOI:** 10.1093/bioadv/vbaf029

**Published:** 2025-02-18

**Authors:** Hajung Kim, Mogan Gim, Seungheun Baek, Soyon Park, Sunkyu Kim, Jaewoo Kang

**Affiliations:** Department of Computer Science and Engineering, Korea University, Seoul 02841, Korea; Department of Biomedical Engineering, Hankuk University of Foreign Studies, Yongin 17035, Korea; Department of Computer Science and Engineering, Korea University, Seoul 02841, Korea; Department of Computer Science and Engineering, Korea University, Seoul 02841, Korea; AIGEN Sciences, Seoul 04778, Korea; Department of Computer Science and Engineering, Korea University, Seoul 02841, Korea; AIGEN Sciences, Seoul 04778, Korea

## Abstract

**Motivation:**

Understanding which genes are significantly affected by drugs is crucial for drug repurposing, as drugs targeting specific pathways in one disease might be effective in another with similar genetic profiles. By analyzing gene expression changes in cells before and after drug treatment, we can identify the genes most impacted by drugs.

**Results:**

The Biologically-Aware Interpretable Differential Gene Expression Ranking (BADGER) model is an interpretable model designed to predict gene expression changes resulting from interactions between cancer cell lines and chemical compounds. The model enhances explainability through integration of prior knowledge about drug targets via pathway information, handles novel cancer cell lines through similarity-based embedding, and employs three attention blocks that mimic the cascading effects of chemical compounds. This model overcomes previous limitations of cell line range and explainability constraints in drug–cell response studies. The model demonstrates superior performance over baselines in both unseen cell and unseen pair split evaluations, showing robust prediction capabilities for untested drug–cell line combinations.

**Availability and implementation:**

This makes it particularly valuable for drug repurposing scenarios, especially in developing therapeutic plans for new or resistant diseases by leveraging similarities with other diseases. All code and data used in this study are available at https://github.com/dmis-lab/BADGER.git.

## 1 Introduction

Identification of molecular mechanisms for cellular drug effects is crucial for improvement in cancer treatments. Advancements in high-throughput sequencing techniques have accelerated pre-clinical studies exploring interactions between various cancer cell lines and potential drug treatments, which led to proliferation of pharmacological data and gene expressions stored in large-scale databases. A notable example of such a database is the LINCS L1000 (Subramanian *et al.* 2017), developed by the Connectivity Map project (Lamb *et al.* 2006).

The availability of LINCS L1000 has motivated researchers to develop computational methods for analyzing the interplay between cancer cell lines and chemical compounds. Prior works focused on predicting drug sensitivity scores using cancer cell lines and chemical compounds as inputs. BiG-DRP (Hostallero *et al.* 2022) learns the relationships between chemical compounds and cell lines using only significant indicators of drug sensitivity.

Our study shifts the spotlight toward predicting the ranking of differentially expressed genes (DEGs). A DEG refers to a gene that shows a change in its expression when comparing two different conditions, such as before and after chemical compound treatment. These changes can be either an increase (up-regulation) or a decrease (down-regulation) in gene expression. By ranking DEGs, we can identify how chemical compounds impact cancer cell lines at the molecular level and spotlight a subset of closely related genes to the effect of chemical compounds, opening doors to understanding related pathways and potential target proteins.

In our research, we concentrate on ranking DEGs within the framework of chemical perturbations. Chemical perturbations refer to the process of altering the normal state of cancer cell lines, through the application of small molecules. Two notable prior works are DeepCE (Pham *et al.* 2021) and CIGER (Pham *et al.* 2022). The authors of DeepCE introduced a deep learning method for predicting gene expressions from cancer cell lines and novel molecular inputs, utilizing a graph neural network for encoding molecules and a multi-head attention mechanism for modeling the relationships between chemical substructures and genes. CIGER, a framework for chemical-induced gene expression ranking, employs similar graph neural networks and attention mechanisms to DeepCE. As the main bottleneck lies in the difficulty of accurately ranking 978 landmark genes based on their differential expression values, both studies explored several learning-to-rank functions to effectively optimize their proposed architecture. These works showed progress in predicting the ranking of DEGs for new compounds with applications in drug repurposing for conditions like COVID-19 and pancreatic cancer.

However, both works feature model architectures that handle a fixed number of cancer cell lines as input, which renders them unable to predict chemical perturbations given novel cancer cell lines. Additionally, they may not adequately account for the intricate relationships between chemical compounds, target proteins, biological pathways, and gene regulatory networks. Moreover, the capability of their commonly used attention mechanism to provide biologically relevant insights is questionable, even though it was originally designed with a focus on model explainability. To address these challenges, we introduce BADGER, a Biologically-Aware interpretable Differential Gene Expression Ranking model. This model is designed to predict the ranking of DEGs given a chemical compound and a cell line as inputs. Upon designing the model, we focus on three main design aspects in this work.

First, BADGER efficiently identifies the effects of any drug compound on any cell line, even those not included in the training dataset. We emphasize the importance of addressing practicability issues, especially when identifying the mechanism of action for new chemical compounds in novel cancer cell lines. Unlike previous models that represent cell lines as one-hot-encoded embeddings, BADGER exploits similarity-based scores derived from basal state gene expressions to build initial representations of cancer cell lines. This method enables the characterization of new cell lines by comparing them to known cell lines from the training data, broadening the coverage of various cancer types in drug repurposing.

Second, the model implements a structured approach to capture the relationships between chemical perturbations and gene expression changes through three specialized attention blocks: (i) Perturbation-Pathway cross attention block modeling potential interactions between chemical compounds and cellular pathways; (ii) Pathway-Gene cross attention block modeling associations between pathways and their related genes; and (iii) Gene–Gene self-attention block modeling potential gene interactions. Together, these blocks provide a framework for analyzing the multiple levels of interactions that may occur during chemical perturbation.

Third, to enhance the explainability of the model’s predictions regarding drug–pathway interactions, we incorporate the attention regularization method proposed from ArkDTA (Gim *et al.* 2023) in the perturbation-pathway attention block. The attention regularization term ensures that the distribution of attention weights—essentially, where the model is “looking” when it makes a prediction—aligns with actual, known drug–pathway relationships. This alignment means the model’s predictions are not just accurate but also based on biologically plausible interactions, which is critical for trust and utility in real-world applications.

Experimental results on ranking DEGs demonstrate BADGER’s robust performance across diverse chemical perturbation scenarios by evaluation under three different dataset split settings: new cell, new drug, and new pair. We further evaluate on external dataset, showing its generalizability. We additionally perform qualitative analysis using BADGER’s attention mechanism and the predicted ranking of DEGs and demonstrate applicability in drug repurposing. Through these results and analyses, we underscore the significant contributions of BADGER.

- BADGER represents a significant advancement in drug efficacy analysis, predicting the impact of any chemical compounds on any cancer cell lines, even those not included in its training set. This approach uses similarity scores based on gene expressions, enabling the model to accurately characterize and adapt to new and diverse cancer cell lines. This feature is particularly critical for the effective repurposing of existing drugs across various cancer types.
- BADGER architecture incorporates three specialized attention blocks to model different aspects of chemical perturbation: the Perturbation-Pathway cross-attention block for modeling potential interactions between compounds and pathways, the Pathway-Gene cross-attention block for modeling relationships between pathways and genes, and the Gene–Gene self-attention block for modeling potential gene associations.
- BADGER integrates the attention regularization method into its perturbation-pathway attention block, a technique introduced in ArkDTA (Gim *et al.* 2023). This aims to align the model’s attention patterns with known drug–pathway interactions to improve interpretability of the predictions. Such an alignment is essential for building confidence and enabling real-world applications, especially in drug repurposing.

## 2 Methods

### 2.1 Dataset

The L1000 database, which contains comprehensive experimental data, was established by exposing cell lines to various small molecules under different dosages and exposure times, and it provides additional information on experimental conditions. We focused on the Level 5 data from L1000, consisting of 720K differential gene expression profiles. These profiles were calculated using the characteristic direction method (Clark *et al.* 2014) across three replicates, comparing gene expression values between control DMSO treatments and corresponding experimental chemical perturbations. To create our chemical perturbation dataset, we applied several preprocessing techniques to the Level 5 data. Consequently, each data instance in our study is defined as a triplet: a small molecule (m∈M), a cell line (c∈C), and the differential expression profile of 978 landmark genes ($Y_{G}$).

As pointed out by the authors of CIGER study, the L1000 database suffers from noisy data issues due to experimental limitations (Pham *et al.* 2022). To alleviate them, we carefully devise data pre-processing strategies for use before building deep learning-based gene ranking models in this study. The following details describe our pre-processing steps, which were applied to an initial set of 720 216 data instances, for constructing the chemical perturbation dataset.

- From the 720 216 data instances, we select those whose chemical perturbations have a dosage of 10 μM and an exposure time of 24 h, following the approach adopted from CIGER study (Pham *et al.* 2022). This selection led to the elimination of 84.15% of the data, leaving 114 156 instances. The dataset is narrowed down to include 11 585 distinct small molecules and 167 unique cell lines.
- From the 114 156 data instances, we remove those that do not pass its Quality Control (QC) introduced by Connectopedia (https://clue.io/connectopedia/l1000_qc). For each data instance to pass the QC, the following requirements should be fulfilled: (i) the cell lines are properly plated and treated, (ii) Polymerase Chain Reaction amplification properly occurs, (iii) beads are properly added throughout the plate, and (iv) any equipment issues are identified and addressed promptly. As a result, 25.71% of the data instances are eliminated, leaving 84 803 instances, comprising 10 335 different small molecules and 165 cell lines.
- From the 84 803 data instances, we remove those with Transcriptional Activity Score (TAS) not exceeding 0.2. TAS is a statistical aggregation of signature strength and replicate correlation and is well known to highlight cell line characteristics depending on its chemical treatment. As a result, 72.40% of data instances are eliminated, leaving a total of 23 409 instances, which include 6737 different small molecules and 114 cell lines.

**Figure 1.**
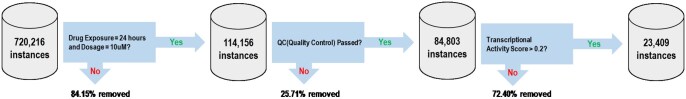
A multi-step filtering process is applied to a dataset based on specific criteria. Initially, the dataset consists of 720 216 instances. By restricting the instances with a drug exposure time of 24 h and a dosage of 10 μM, 114 156 instances are left. Further quality control measures are then applied, leaving 84 803 instances. Finally, by selecting only those instances with a transcriptional activity score >0.2, the dataset is refined to 23 409 instances.

Figure 1 outlines the pre-processing steps used to construct our dataset. It is important to note that while our dataset and model experiments primarily utilize 6737 small molecules (|M|=6737) and 114 cell lines (|C|=114), BADGER is also capable of predicting the ranking of DEGs for chemical perturbations involving out-of-dataset small molecules and/or cell lines.

In our study, we analyze the connections between small molecules and their target-associated pathways using data from two sources: the L1000 database and the Molecular Signatures Database (MSigDB). The L1000 dataset provides the target information about the small molecules, while MSigDB offers the Human Collection pathways. Among the vast array of pathways annotated in MSigDB, we focus on 140 pathways overlapping with the landmark genes. A pathway is marked as “positive” if it is linked to the target of the small molecule, and as “negative” if there is no such association. This relationship is represented in a binary vector $Y_{P}\in {\left[0,1\right]}^{\left|P\right|}$, which uses 1 s and 0 s to represent “positive” and “negative” associations, respectively. This binary vector provides a detailed map of how each molecule influences various biological pathways.

### 2.2 Model architecture

#### 2.2.1 Overview

**Figure 2.**
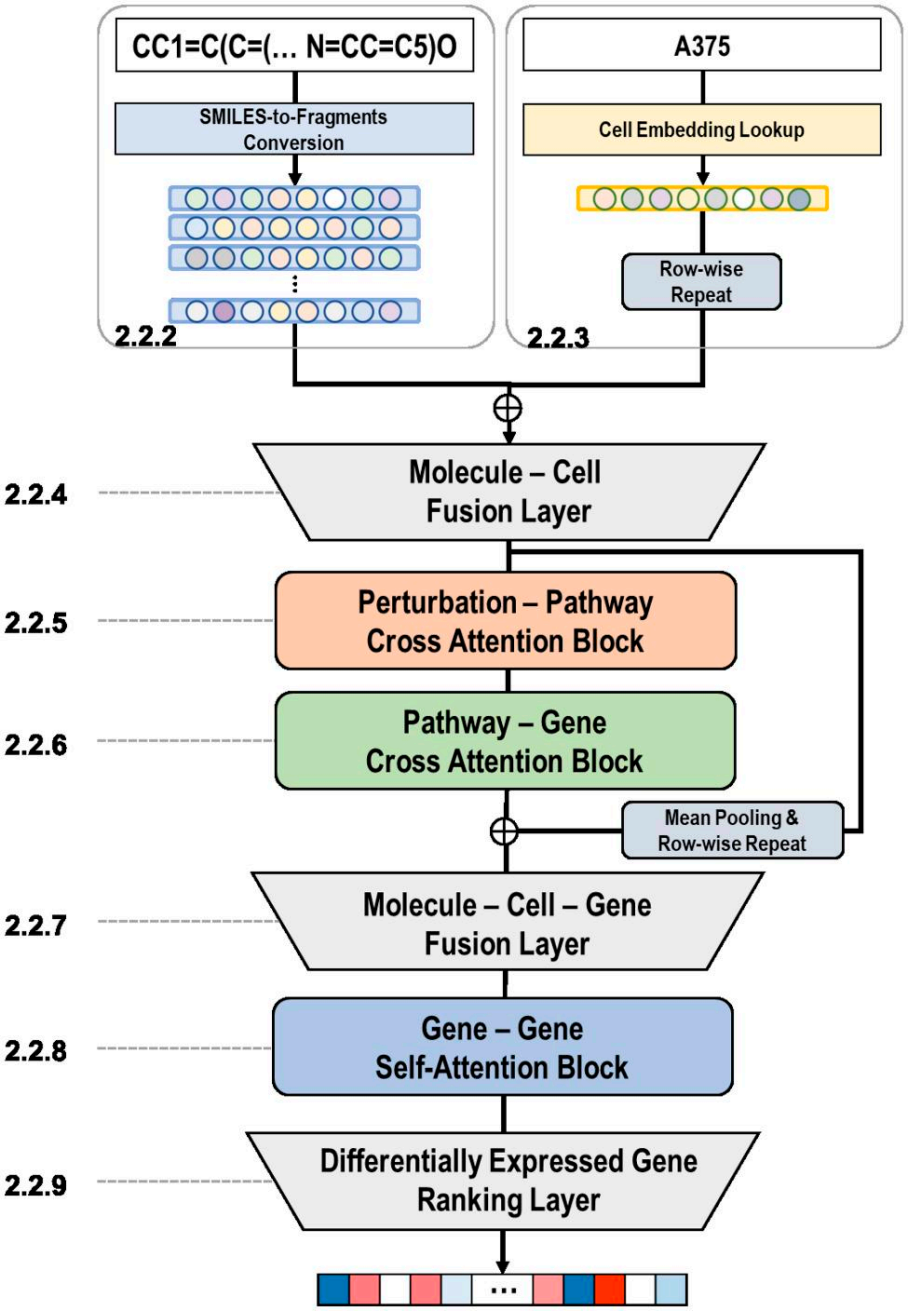
Overview of BADGER. The model takes as its input a pair consisting of a small molecule and a cell line. This input pair undergoes an integration and refinement process through fusion layers, followed by a sequence of three specialized attention blocks: the Perturbation-Pathway Cross Attention Block (highlighted in orange), the Pathway-Gene Cross Attention Block (highlighted in green), and the Gene-Gene Self Attention Block (highlighted in blue). The concluding stage of the model is the gene expression ranking layer, which ranks 978 landmark genes. This ranking effectively represents the changes in gene expression that occur as a result of the chemical perturbation.

BADGER takes a small molecule and a cancer cell line as inputs and predicts its differential expression profiles of 978 landmark genes as output. Its architecture comprises modality-wise encoders (Molecule Encoding Layer, Cell Encoding Layer), modality-fusion layers (Molecule-Cell Fusion Layer, Molecule-Cell-Gene Fusion Layer), a series of attention blocks (Perturbation-Pathway Cross-Attention Block, Pathway-Gene Cross-Attention Block, and Gene-Gene Self-Attention Block), and a Differentially Expressed Gene Ranking Layer consisting 978 gene-wise DEG ranking layers. The definition for BADGER is mathematically expressed as follows:

(1) $${\hat{Y}}_{G}=\text{BADGER}(\text{M},\;\text{C})$$

where m and c refer to input a small molecule and a cancer cell line respectively while ${\hat{Y}}_{G}\in R^{978}$ are differential expression profiles of 978 landmark genes. Figure 2 illustrates BADGER’s overall model architecture.

#### 2.2.2 Molecule encoding layer

The Molecule Encoding Layer transforms a small molecule into a set of molecular fragment embeddings. The initial representation for the input small molecule is its SMILES string and is converted into a set of molecular fragments via the BRICS (Degen *et al.* 2008) algorithm imported from RDKit library. The decomposed fragments are known to have distinguishable chemical and pharmaceutical characteristics. Each fragment is represented as a 1024-dimensional Morgan Fingerprint and is subsequently propagated through a MLP with non-linear activation and dropout. The Molecule Encoding Layer that takes the SMILES representation of small molecule m as input is mathematically expressed as follows:

$$\begin{array}{c} F=S2M(\text{BRICS}(\text{M}))\\ X_{M}=\mathrm{MLP}(F) \end{array}$$

where BRICS is the BRICS algorithm for decomposing the input molecule into *k* fragments, S2M is the SMILES-to-Morgan Fingerprint conversion process applied to all fragments, $F\in {\left[0,1\right]}^{k\times 1024}$ is a set of *k* 1024-dimensional fragment-wise Morgan fingerprints, and $X_{M}\in R^{k\times d}$ is a set of *k d*-dimensional molecular fragment embeddings.

#### 2.2.3 Cell encoding layer

The Cell Encoding Layer transforms a cancer cell line into a single *d*-dimensional embedding. We employ a similarity-based representation method by calculating similarity scores between the input cell line and all 114 cell lines available in our dataset. The cell similarity scores are determined using Spearman’s rank correlation coefficient, which measures the similarity based on their basal state gene expressions. As a result, the initial representation for the input cell line is a 114-dimensional similarity-based vector and is subsequently propagated through a MLP with non-linear activation and dropout. The Cell Encoding Layer that takes a cell line c as input is mathematically expressed as follows:

(2) $$c_{\mathrm{sim}}=\left[\begin{array}{cccc} \text{sim}(c,c_{1}) & \text{sim}(c,c_{2}) & \cdots & \text{sim}(c,c_{114}) \end{array}\right]$$

 

(3) $$x_{C}=\mathrm{MLP}(c_{\mathrm{sim}})$$

where through calculation of similarity, the input cell expression c is transformed into a 114-dimensional initial cell embedding $c_{\mathrm{sim}}\in R^{114},\;x_{C}\in R^{d}$ is a *d*-dimensional cell line embedding.

#### 2.2.4 Molecule-Cell fusion layer

The Molecule-Cell Fusion Layer fuses both encoded modality-wise embeddings $X_{M}$ and $x_{C}$ into a set of perturbation embeddings $X_{M+C}$. The main intuition is to augment the base chemical information with cell-specific characteristics that may influence the underlying cellular pathways. In this layer, $x_{C}$ is repeatedly expanded into row-repeated $X_{C}\in R^{k\times d}$ and is concatenated with $X_{M}$. The concatenated embeddings are then propagated through a MLP with non-linear activation and dropout to output a set of perturbation embeddings. The Molecule-Cell Fusion Layer that takes $X_{M}$ and $X_{C}$ as input is mathematically expressed as follows:

(4) $$X_{C}={\left[1,1,\ldots ,1\right]}^{T}⊗\;x_{C}$$

 

(5) $$X_{M+C}=\mathrm{MLP}(X_{M}\;⊕\;X_{C})$$

where |[1,1,…,1]|=k refers to row-wise repetition of $x_{C}$ that expands it to *k* identical embeddings, $X_{M+C}\in R^{k\times d}$ a set of *k d*-dimensional “molecular fragment-wise” perturbation embeddings.

#### 2.2.5 Perturbation-Pathway Cross-Attention Block

**Figure 3.**
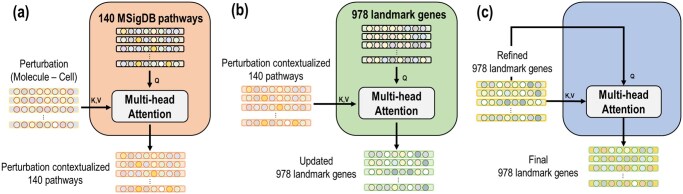
Detailed description of three attention blocks (a–c). (a): The input key-value is perturbation embeddings while the query is a 140 pathway embeddings from MSigDB appended with a pseudo-embedding $\vec{p}$. The output of this block is a perturbation contextualized pathway embeddings. (b): The input key-value is the output of (a) and the query is embeddings of 978 landmark genes. The output of this block is updated gene embeddings. (c): The input key-query-value is refined gene embeddings. The output represents 978 landmark gene embeddings, which go through the final differential gene expression ranking prediction layer.

The Perturbation-Pathway Cross-Attention Block employs a multi-head cross-attention mechanism between two different modalities: molecular fragments and pathways. Each pathway is assigned scores based on predefined terms from MSigDB. In a similar way to how we process cell embeddings, we compute the Spearman’s correlation for every pair of pathways. As shown in Fig. 3a, it takes a set of *k d*-dimensional perturbation embeddings $X_{M+C}$ as key and value inputs and its internal set of 140 trainable, shareable pathway embeddings **P** as query input to output a set of 140 perturbation-contextualized pathway embeddings $X_{P}$.

General cross-attention mechanism involves calculation of pairwise attention weights between pathways (queries) and fragment-wise perturbations (keys). For each pathway, the attention weights are distributed to the fragment-wise perturbations regardless of drug–pathway associations. To address this, we employ ArkDTA’s attention regularization method by first appending a randomly initialized pseudo-embedding to the current set of fragment-wise perturbations (Gim *et al.* 2023). This method coerces the Perturbation-Pathway Cross-Attention Block to concentrate its distribution of attention weights to the pseudo-embedding when there is no drug-pathway association. Detailed explanation for this method is available in Gim *et al.*

The Perturbation-Pathway Cross-Attention Block mathematically notated as **PPAB** that takes a set of perturbation embeddings $X_{M+C}$ as input is mathematically expressed as follows:

(6) $$X_{M+C}^{+}=X_{M+C}\cup \left\{p_{F}\right\}$$

 

(7) $$X_{P}=\mathrm{PPAB}(X_{M+C}^{+})$$

where $X_{M+C}^{+}\in R^{(k+1)\times d}$ is a set of *k *+* *1 *d*-dimensional fragment-level perturbation embeddings including pseudo-fragment $p_{F},\;X_{P}\in R^{140\times d}$ is a set of 140 *d*-dimensional *perturbation-contextualized* pathway embeddings. More implementation details for the **PPAB** are available in Appendix 1.

#### 2.2.6 Pathway-Gene Cross-Attention Block

The Pathway-Gene Cross-Attention Block employs multi-head cross-attention mechanism between pathways and landmark genes. As shown in Fig. 3b, it takes as set of 140 *d*-dimensional perturbation-contextualized pathway embeddings $X_{P}$ as key, value input, and its internal set of 978 trainable, shareable landmark gene embeddings **G** as query to output a set of 978 updated landmark gene embeddings $X_{G}$. The pathway embeddings are initialized based on predefined terms from the Gene Ontology (GO) (Ashburner *et al.* 2000). The landmark gene embeddings are derived from predefined terms from the Gene2Vec (Du *et al.* 2019).

The Pathway-Gene Cross-Attention Block share similar design aspects with the Perturbation-Pathway Cross-Attention Block but do not employ attention regularization. The Pathway-Gene Cross-Attention Block mathematically notated as “PGAB” that takes a set of chemical-induced pathway embeddings $X_{P}$ as input is mathematically expressed as follows:

(8) $$X_{G}=\mathrm{PGAB}(X_{P})$$

where $X_{G}\in R^{978\times d}$ is a set of 978 *d*-dimensional updated landmark gene embeddings.

#### 2.2.7 Molecule-Cell-Gene Fusion Layer

The Molecule-Cell-Gene Fusion Layer combines the updated gene embeddings $X_{G}$ with the previously used perturbation embeddings $X_{M+C}$, which contain a mixture of chemical and cell characteristics. The main purpose of this layer is to preserve the small molecule and cell information in latent space. In this layer, $X_{M+C}$ is first pooled mean-wise into a single embedding $x_{M+C}\in R^{d}$. The pooled embedding is then repeatedly expanded into row-repeated $X_{M+C}^{*}\in R^{978\times d}$ and is concatenated with $X_{G}$. The concatenated embeddings are then propagated through a MLP with non-linear activation and dropout to output a set of refined 978 landmark gene embeddings. The Molecule-Cell-Gene Fusion Layer that takes $X_{M+C}$ and $X_{G}$ as input is mathematically expressed as follows:

(9) $$X_{M+C}^{*}={\left[1,1,\ldots ,1\right]}^{T}⊗\mathrm{MeanPool}(X_{M+C})$$

 

(10) $$X_{G}^{*}=\mathrm{MLP}(X_{G}\;⊕\;X_{M+C}^{*})$$

where $X_{G}^{*}\in R^{978\times d}$ a set of 978 *d*-dimensional refined gene embeddings.

#### 2.2.8 Gene–Gene Self-Attention Block

The Gene-Gene Self-Attention Block employs multi-head self-attention mechanism within the updated landmark gene embeddings to enrich them with gene–gene pairwise relationship features. The output of this module is a set of final 978 landmark gene embeddings $X_{Z}$. The Gene-Gene Self-Attention Block mathematically notated as **GGAB** that takes a set of updated landmark gene embeddings $X_{G}^{*}$ as input is mathematically expressed as follows:

(11) $$X_{Z}=\mathrm{GGAB}(X_{G}^{*})$$

where $X_{Z}\in R^{978\times d}$ is a set of 978 *d*-dimensional final state landmark gene embeddings.

#### 2.2.9 Differentially Expressed Gene Ranking Layer

The Differentially Expressed Gene Ranking Layer contains 978 feed-forward layers where each of them takes its corresponding final 978 landmark gene embedding as input and predicts its differential gene expression value. Contrary to the previous model architectures DeepCE (Pham *et al.* 2021) and CIGER (Pham *et al.* 2022), which uses a single feedforward layer for predicting the ranking of DEGs, BADGER employs gene-specific ranking layers to effectively leverage the individual characteristics of landmark genes. For each *i*th gene $g_{i}\in G$, the Differentially Expressed Gene Ranking Layer predicts its corresponding differential expression value ${\hat{y}}_{i}\in G$ given its final embedding $x_{i}\in X_{Z}$ as input, which is mathematically expressed as follows:

(12) $${\hat{y}}_{i}=\mathrm{FeedForwar}d_{i}(x_{i})$$

where $\mathrm{FeedForwar}d_{i}$ is the *i*th feed-forward layer that outputs the differential expression value ${\hat{y}}_{i}\in R$ for an individual landmark gene $g_{i}\in G$.

### 2.3 Training and optimization

We train BADGER for maximum of 100 epochs using the Adam optimizer with its learning rate and batch size set to 0.0001 and 64, respectively. The loss objective for training BADGER consists of two terms, which are the main ranking loss term $L_{1}$ and attention regularization term $L_{2}$. For the main ranking loss term, we employed a hybrid loss criterion, which is a summation of two learning-to-rank loss functions RankCosine (Qin *et al.* 2008) and Mean Squared Error (MSE). The attention regularization term was adopted from ArkDTA to provide better model explainability related to drug–pathway relationships (Gim *et al.* 2023). The attention regularization term enforces the overall distribution attention weights extracted from the “PPAB” to be aligned with the actual drug–pathway relationship ground truth labels.

The instance-wise ranking loss term for training BADGER is mathematically expressed as follows:

(13) $$L_{1}=\mathrm{RankCosine}({\hat{Y}}_{G},Y_{G})+\mathrm{MSE}({\hat{Y}}_{G},Y_{G})$$

 

(14) $${\hat{Y}}_{G}=[{\hat{y}}_{1},{\hat{y}}_{2}\cdots {\hat{y}}_{978}]$$

 

(15) $$Y_{G}=[y_{1},y_{2}\cdots y_{978}]$$

where ${\hat{Y}}_{G}$ is a list of 978 predicted landmark gene expression values, $Y_{G}$ is a list of 978 actual expression values, *RankCosine* is a loss function based on cosine similarity between two ranked lists, and *MSE* is mean squared error function.

In BADGER’s “PPAB,” the attention weights are obtained by averaging the matrices from the Multihead Attention Layer, resulting in a matrix $A\in R^{140\times (k+1)}$. These weights are calculated using 140 pathway embeddings as queries and *k* perturbation embeddings appended with a pseudo-embedding as keys. The sum of the first *k* attention weights corresponding to actual perturbations embeddings for each pathway in **A** represents the probability score of a small molecule m being associated with a pathway. If the molecule is actually associated with target-associated pathway, the score is forced to be maximized toward 1. On the contrary, if the molecule is irrelevant to the pathway, the score is forced to be minimized toward 0.

The instance-wise auxiliary loss term for attention regularization of BADGER is mathematically expressed as follows:

(16) $$L_{2}=\mathrm{CrossEntropy}(Y_{P},{\hat{Y}}_{P})$$

where $Y_{P}$ represents a binary vector of actual drug–pathway association label, while ${\hat{Y}}_{P}$ indicates a binary vector of predicted drug–pathway association label.

The whole instance-wise auxiliary loss term for training BADGER is $L=L_{1}+\alpha L_{2}$ where the attention regularization coefficient α is a hyperparameter set to 0.05.

## 3 Results

### 3.1 Experiments on the L1000 dataset

We conducted a comprehensive experiment using a newly constructed L1000-based chemical perturbation dataset and compared BADGER with various baseline methods ranging from simple approaches to complex deep learning approaches. For simpler baselines, we implement: (i) AverageRank, a naive approach that creates a reference ranking by averaging gene expression values across the training dataset and comparing it with test set rankings, and (ii) a k-Nearest Neighbors (kNN) regressor that utilizes the learned embedding space to make predictions based on the most similar cases (*k* = 5). For more sophisticated deep learning-based approaches, we compared against: (i) a Multi-Layer Perceptron (MLP), (ii) DeepCE (Pham *et al.* 2021), and (iii) CIGER (Pham *et al.* 2022). The MLP consists of multiple fully connected layers with non-linear transformations to learn complex patterns in the data. Both kNN and MLP process the concatenated representations from ChemBERTa-based drug embeddings and similarity-based cell line features through a fusion layer.

The models are evaluated under three dataset split settings—(i) New Cell line: cell lines have unseen, (ii) New Drug: drugs have unseen, and (iii) New Pair: both cell and drug have unseen—to assess their generalizability in predicting responses to new cell lines, drugs, and cell line–drug pairs. This is done using a five-fold cross-validation approach. Two evaluation metrics, Normalized Discounted Cumulative Gain and Precision at K (P@K), are used to evaluate the ranking results for DEGs. For P@K calculation, we followed the evaluation approach established in the baseline work (Pham *et al.* 2021). The metric calculates how many top-K predicted genes appear within the ground truth’s top-200 DEGs. Specifically, the positive P@K measures the overlap between our model’s top-K up-regulated gene predictions and the ground truth’s top-200 up-regulated genes. Similarly, the negative P@K measures the overlap between predicted top-K down-regulated genes and the ground truth’s top-200 down-regulated genes.

Table 1 shows that BADGER slightly underperforms in the New Drug setting for ranking up-regulated genes but performs significantly better in the New Cell and New Pair settings compared to the baseline models. The improved performance in the New Cell setting is attributed to BADGER’s use of a similarity-based cell embedding strategy, which provides a more detailed representation of cell line characteristics, leading to enhanced generalizability across different cancer cell types. Overall, BADGER exhibits robustness in ranking DEGs given various chemical perturbation contexts.

**Table 1.** Results of experiments on the L1000 dataset.^a^

Model	Up-regulated Gene Ranking					Down-regulated Gene Ranking				
	NDCG	P@10	P@50	P@100	P@200	NDCG	P@10	P@50	P@100	P@200
**Split method: New Cell line**										
**AverageRank**	$69.82^{\;\pm \;0.81}$	$37.47^{\;\pm \;3.00}$	$33.57^{\;\pm \;2.28}$	$31.27^{\;\pm \;2.13}$	$29.03^{\;\pm \;1.43}$	$72.42^{\;\pm \;1.18}$	$45.79^{\;\pm \;3.90}$	$39.61^{\;\pm \;3.45}$	$36.57^{\;\pm \;3.02}$	$32.28^{\;\pm \;2.18}$
**kNN**	$71.93^{\;\pm \;2.50}$	$42.88^{\;\pm \;8.04}$	$37.60^{\;\pm \;6.84}$	$34.60^{\;\pm \;5.77}$	$31.00^{\;\pm \;4.23}$	$72.74^{\;\pm \;2.28}$	$44.14^{\;\pm \;6.79}$	$39.81^{\;\pm \;6.29}$	$36.83^{\;\pm \;5.62}$	$32.63^{\;\pm \;4.32}$
**MLP**	$73.20^{\;\pm \;2.35}$	$46.90^{\;\pm \;8.22}$	$40.93^{\;\pm \;7.01}$	$37.19^{\;\pm \;5.70}$	$32.92^{\;\pm \;4.10}$	$72.63^{\;\pm \;1.82}$	$42.61^{\;\pm \;7.74}$	$38.51^{\;\pm \;3.85}$	$36.36^{\;\pm \;3.65}$	$32.77^{\;\pm \;2.95}$
**DeepCE (Pham *et al.* 2021)**	$73.28^{\;\pm \;1.4}$	$46.99^{\;\pm \;4.6}$	$41.61^{\;\pm \;4.3}$	$38.25^{\;\pm \;3.7}$	$33.58^{\;\pm \;2.7}$	$71.46^{\;\pm \;1.2}$	$41.70^{\;\pm \;5.0}$	$36.50^{\;\pm \;3.2}$	$33.65^{\;\pm \;2.4}$	$30.51^{\;\pm \;1.7}$
**CIGER (Pham *et al.* 2022)**	$71.28^{\;\pm \;1.6}$	$41.02^{\;\pm \;5.6}$	$35.93^{\;\pm \;4.0}$	$32.74^{\;\pm \;3.2}$	$29.60^{\;\pm \;2.3}$	$71.50^{\;\pm \;1.5}$	$41.60^{\;\pm \;6.0}$	$36.00^{\;\pm \;4.6}$	$32.86^{\;\pm \;3.7}$	$29.65^{\;\pm \;2.6}$
**BADGER** (Ours)	** $74.80^{\;\pm \;1.8}$ **	** $54.11^{\;\pm \;6.0}$ **	** $45.85^{\;\pm \;5.5}$ **	** $40.84^{\;\pm \;4.6}$ **	** $35.34^{\;\pm \;3.3}$ **	** $75.15^{\;\pm \;1.7}$ **	** $53.55^{\;\pm \;5.3}$ **	** $46.69^{\;\pm \;4.9}$ **	** $42.43^{\;\pm \;4.2}$ **	** $36.79^{\;\pm \;3.2}$ **
**Split method: New Drug**										
**AverageRank**	$69.86^{\;\pm \;0.26}$	$37.73^{\;\pm \;0.77}$	$33.35^{\;\pm \;0.53}$	$31.29^{\;\pm \;0.46}$	$28.94^{\;\pm \;0.31}$	$72.35^{\;\pm \;0.18}$	$45.45^{\;\pm \;0.50}$	$39.34^{\;\pm \;0.30}$	$36.32^{\;\pm \;0.33}$	$32.10^{\;\pm \;0.24}$
**kNN**	$70.52^{\;\pm \;0.18}$	$37.13^{\;\pm \;0.60}$	$32.92^{\;\pm \;0.46}$	$30.68^{\;\pm \;0.38}$	$28.17^{\;\pm \;0.31}$	$71.70^{\;\pm \;0.20}$	$40.81^{\;\pm \;0.38}$	$36.72^{\;\pm \;0.44}$	$33.98^{\;\pm \;0.43}$	$30.46^{\;\pm \;0.31}$
**MLP**	$72.65^{\;\pm \;0.25}$	$43.68^{\;\pm \;0.99}$	$37.85^{\;\pm \;0.70}$	$34.78^{\;\pm \;0.54}$	$31.48^{\;\pm \;0.37}$	$72.52^{\;\pm \;0.27}$	$40.93^{\;\pm \;2.03}$	$38.04^{\;\pm \;0.59}$	$36.10^{\;\pm \;0.25}$	$32.64^{\;\pm \;0.23}$
**DeepCE (Pham *et al.* 2021)**	$75.01^{\;\pm \;1.2}$	$52.31^{\;\pm \;4.1}$	$43.77^{\;\pm \;2.9}$	$39.30^{\;\pm \;2.2}$	$34.60^{\;\pm \;1.5}$	$71.68^{\;\pm \;0.2}$	$40.50^{\;\pm \;1.1}$	$35.51^{\;\pm \;0.6}$	$32.97^{\;\pm \;0.5}$	$30.21^{\;\pm \;0.4}$
**CIGER (Pham *et al.* 2022)**	** $75.93^{\;\pm \;0.1}$ **	** $54.85^{\;\pm \;0.4}$ **	** $48.29^{\;\pm \;0.4}$ **	** $43.92^{\;\pm \;0.4}$ **	** $38.00^{\;\pm \;0.2}$ **	$74.03^{\;\pm \;0.3}$	$48.49^{\;\pm \;1.4}$	$41.22^{\;\pm \;0.7}$	$37.27^{\;\pm \;0.5}$	$33.08^{\;\pm \;0.3}$
**BADGER** (Ours)	$73.73^{\;\pm \;0.3}$	$48.17^{\;\pm \;0.9}$	41.38 ± 0.8	$37.51^{\;\pm \;0.8}$	$33.14^{\;\pm \;0.6}$	** $74.88^{\;\pm \;0.3}$ **	** $51.69^{\;\pm \;1.0}$ **	** $45.50^{\;\pm \;1.0}$ **	** $41.51^{\;\pm \;0.9}$ **	** $36.08^{\;\pm \;0.7}$ **
**Split method: New Pair (new cell line and new drug)**										
**AverageRank**	$69.78^{\;\pm \;0.86}$	$37.85^{\;\pm \;3.04}$	$33.36^{\;\pm \;2.04}$	$31.10^{\;\pm \;1.89}$	$29.00^{\;\pm \;1.28}$	$72.48^{\;\pm \;1.07}$	** $45.87^{\;\pm \;4.11}$ **	$39.76^{\;\pm \;3.69}$	$36.67^{\;\pm \;3.03}$	$32.38^{\;\pm \;2.19}$
**kNN**	$68.80^{\;\pm \;0.72}$	$31.57^{\;\pm \;1.90}$	$29.22^{\;\pm \;1.87}$	$27.72^{\;\pm \;1.54}$	$26.01^{\;\pm \;1.21}$	$70.20^{\;\pm \;0.89}$	$35.55^{\;\pm \;2.81}$	$32.70^{\;\pm \;2.51}$	$30.59^{\;\pm \;2.18}$	$27.96^{\;\pm \;1.76}$
**MLP**	$71.14^{\;\pm \;1.04}$	$39.51^{\;\pm \;4.01}$	$34.75^{\;\pm \;2.82}$	$32.22^{\;\pm \;2.11}$	$29.59^{\;\pm \;1.62}$	$70.56^{\;\pm \;1.39}$	$29.86^{\;\pm \;4.86}$	$33.22^{\;\pm \;4.80}$	$33.35^{\;\pm \;3.51}$	$30.92^{\;\pm \;2.38}$
**DeepCE (Pham *et al.* 2021)**	$70.95^{\;\pm \;0.8}$	$39.61^{\;\pm \;4.1}$	$34.59^{\;\pm \;1.8}$	$32.13^{\;\pm \;1.3}$	$29.57^{\;\pm \;1.0}$	$70.97^{\;\pm \;0.9}$	$39.73^{\;\pm \;0.4}$	$34.82^{\;\pm \;0.2}$	$32.37^{\;\pm \;1.5}$	$29.70^{\;\pm \;1.1}$
**CIGER (Pham *et al.* 2022)**	$71.31^{\;\pm \;1.1}$	$40.09^{\;\pm \;3.4}$	$34.94^{\;\pm \;2.4}$	$32.18^{\;\pm \;1.8}$	$29.40^{\;\pm \;1.3}$	$71.13^{\;\pm \;1.0}$	$39.37^{\;\pm \;3.4}$	$34.31^{\;\pm \;2.8}$	$31.64^{\;\pm \;2.5}$	$29.01^{\;\pm \;2.0}$
**BADGER **(Ours)	** $71.55^{\;\pm \;1.6}$ **	** $41.70^{\;\pm \;6.5}$ **	** $36.70^{\;\pm \;5.0}$ **	** $33.79^{\;\pm \;4.0}$ **	** $30.40^{\;\pm \;2.8}$ **	** $72.84^{\;\pm \;1.4}$ **	$45.70^{\;\pm \;5.5}$	** $40.08^{\;\pm \;4.3}$ **	** $36.77^{\;\pm \;3.6}$ **	** $32.60^{\;\pm \;2.8}$ **

a The model’s ability to rank up-regulated and down-regulated genes is evaluated using a five-fold cross-validation approach, employing different data splitting methods. Two key metrics are used: “Normalized Discounted Cululative Gain” (NDCG) and “Precision at K” (P@K). A higher NDCG value indicates a more accurate ordering of genes, while a higher P@K score reflects more precise identification of the correct K genes from a set of 200 up- or down-regulated genes. The standard deviation observed across the five-folds is denoted by the superscript accompanying each score. Bold indicates the highest score among all models.

It is important to acknowledge the limitations in BADGER’s performance in the New Drug setting, particularly for ranking up-regulated genes. Upon further analysis, two key factors have been identified: dataset imbalance and the learning of generic up-regulation patterns. Specifically, the dataset contains 98 871 fewer up-regulated genes compared to down-regulated genes, resulting in a significant imbalance that may have hindered the model’s ability to effectively learn up-regulation patterns. Furthermore, data analysis revealed that certain genes consistently appeared in the top rankings regardless of the specific drug, suggesting that the model may have learned generic up-regulation patterns rather than drug-specific ones.

These factors may explain why BADGER underperforms relative to baseline models in predicting up-regulated genes for new drugs. The data imbalance likely caused the model to focus more on down-regulation patterns, which were more prevalent in the training data. Additionally, by learning generic up-regulation patterns, the model may have predicted the same set of genes to be up-regulated across multiple drugs, thereby failing to capture drug-specific up-regulation effects. As a result, when confronted with novel drugs in the test set, the model may have relied on these generic patterns, leading to reduced accuracy in identifying drug-specific up-regulated genes.

These limitations become more apparent when examining model performance across different architectures. For down-regulated genes, the baseline models generally underperform compared to the average method across all split settings (New Drug, New Cell Line, and New Pair). BADGER shows competitive performance across New Drug and New Cell Line splits but fails to outperform the AverageRank baseline in the New Pair split. This pattern stands in contrast to the up-regulated genes scenario, where deep learning models consistently demonstrate superior performance over the AverageRank baseline. The complex models’ varying performance between up- and down-regulated genes suggests that these two biological processes might have fundamentally different patterns of complexity. While deep learning architectures can effectively capture and generalize patterns for up-regulated genes, their sophisticated modeling capacity does not translate to improved performance for down-regulated genes. This is particularly evident in the New Pair split, where even BADGER, despite its architectural sophistication, struggles to outperform the simple average baseline. The fact that simpler approaches work better for down-regulated genes across most experimental settings suggests that gene down-regulation might follow more straightforward patterns where complex architectural modeling offers minimal advantages.

### 3.2 External evaluation on sci-Plex dataset

The foundational dataset used in this research is L1000, of which its gene expressions were measured using the microarray technique (Bumgarner 2013). To evaluate BADGER’s generalizability, we perform additional evaluation on an external dataset sci-Plex. sci-Plex is a collection of gene expressions measured by a more advanced approach, RNA-sequencing, specifically tailored for pooled single-nucleus transcriptomic profiling. This method is particularly applied to three distinct cell lines: A549, MCF7, and K562 (Srivatsan *et al.* 2020). The sci-Plex dataset includes the exposure of cells to 1 of 188 different compounds, in addition to a DMSO vehicle control. This comprehensive array of compounds allows for a robust assessment of the model’s predictive capabilities across a diverse chemical landscape.

Table 2 shows the external evaluation results comparing BADGER’s predictability with other baselines. For the Sci-Plex test, we used a checkpoint from the model trained on our dataset, ensuring that the Sci-Plex data was unseen during training. BADGER is highlighted in bold for its scores, indicating that it outperforms the other two models across all metrics in both the up-regulated and down-regulated gene ranking. The results emphasizes the generalizability and reliability in predicting the ranking of DEGs in the context of new pair.

**Table 2.** Results of the external evaluation on sci-Plex dataset.^a^

Model	Up-regulated Gene Ranking					Down-regulated Gene Ranking				
	NDCG	P@10	P@50	P@100	P@200	NDCG	P@10	P@50	P@100	P@200
**Split method: new cell line and new drug**										
**DeepCE** (Pham *et al.* 2021)	$69.21^{\;\pm \;0.2}$	$31.08^{\;\pm \;0.8}$	$26.21^{\;\pm \;0.7}$	$24.80^{\;\pm \;0.6}$	$23.34^{\;\pm \;0.4}$	$67.82^{\;\pm \;0.1}$	$30.37^{\;\pm \;0.4}$	$26.47^{\;\pm \;0.5}$	$24.65^{\;\pm \;0.4}$	$22.83^{\;\pm \;0.2}$
**CIGER** (Pham *et al.* 2022)	$69.41^{\;\pm \;0.6}$	$32.30^{\;\pm \;1.9}$	$27.39^{\;\pm \;1.3}$	$25.43^{\;\pm \;1.0}$	$23.61^{\;\pm \;0.7}$	$67.73^{\;\pm \;0.2}$	$29.62^{\;\pm \;2.0}$	$26.22^{\;\pm \;0.6}$	$24.29^{\;\pm \;0.3}$	$22.60^{\;\pm \;0.3}$
**BADGER** (Ours)	** $70.43^{\;\pm \;0.4}$ **	** $34.85^{\;\pm \;2.1}$ **	** $29.53^{\;\pm \;1.2}$ **	** $27.35^{\;\pm \;0.9}$ **	** $25.21^{\;\pm \;0.6}$ **	** $68.33^{\;\pm \;0.3}$ **	** $30.67^{\;\pm \;1.2}$ **	** $27.97^{\;\pm \;0.7}$ **	** $25.83^{\;\pm \;0.7}$ **	** $23.79^{\;\pm \;0.7}$ **

a The model’s performance in identifying up-regulated and down-regulated genes was assessed through five-fold cross-validation, using various data splits (new cell, new drug, new pair) and two key metrics: “Normalized Discounted Cumulative Gain” (NDCG) and “Precision at K” (P@K). The superscript values represent the standard deviation. Bold indicates the highest score among all models.

### 3.3 Ablation study

To further evaluate the contribution of our proposed perturbation-pathway cross-attention block, we conducted an ablation study. This study aims to quantify the impact of this novel component on our model’s performance and to provide empirical evidence for its significance in capturing important biological interactions. Our baseline models utilize attention mechanisms based on perturbation–gene and gene–gene interactions. The key innovation in our proposed model is the introduction of an additional cross-attention mechanism for perturbation-pathway interactions, which serves as a bridge between the other interactions. To isolate the effect of this component, we compared our full model against a variant where the perturbation-pathway cross-attention block was removed.

Table 3 presents the results of our ablation study, comparing the performance of our full model against the variant without the perturbation-pathway cross-attention block. The results clearly demonstrate the significant impact of the perturbation-pathway cross-attention block on our model’s performance. Without this component, we observe a notable decrease in all metrics, highlighting its crucial role in capturing important biological interactions and improving prediction accuracy.

**Table 3.** Ablation study comparing BADGER with its variant without Perturbation-Pathway Attention Block (PPAB).^a^

Model	Up-regulated Gene Ranking					Down-regulated Gene Ranking				
	NDCG	P@10	P@50	P@100	P@200	NDCG	P@10	P@50	P@100	P@200
**Split method: New Cell line**										
wo PPAB	73.59^ ± 1.5^	49.41^ ± 5.8^	41.67^ ± 4.3^	37.57^ ± 3.4^	33.16^ ± 2.5^	74.42^ ± 1.6^	51.16^ ± 5.0^	44.54^ ± 4.3^	40.60^ ± 3.8^	35.42^ ± 2.8^
BADGER	**74.80 ** ^±** **1.8^	**54.11 ** ^±** **6.0^	**45.85 ** ^±** **5.5^	**40.84 ** ^±** **4.6^	**35.34 ** ^±** **3.3^	**75.15 ** ^±** **1.7^	**53.55 ** ^±** **5.3^	**46.69 ** ^±** **4.9^	**42.43 ** ^±** **4.2^	**36.79 ** ^±** **3.2^
**Split method: New Drug**										
wo PPAB	71.78 ^± 0.2^	41.08 ^± 1.3^	35.73 ^± 0.7^	33.00 ^± 0.4^	30.01 ^± 0.2^	73.21^ ± 0.1^	46.10^ ± 0.9^	40.74^ ± 0.6^	37.41^ ± 0.5^	33.06^ ± 0.4^
BADGER	**73.73 ** ^±** **0.3^	**48.17 ** ^±** **0.9^	**41.38 ** ^±** **0.8^	**37.51 ** ^±** **0.8^	**33.14 ** ^±** **0.6^	**74.88 ** ^±** **0.3^	**51.69 ** ^±** **1.0^	**45.50 ** ^±** **1.0^	**41.51 ** ^±** **0.9^	**36.08 ** ^±** **0.7^
**Split method: New Pair (new cell line and new drug)**										
wo PPAB	70.77 ^± 1.3^	38.29 ^± 4.9^	33.71 ^± 4.0^	31.44 ^± 3.2^	28.96 ^± 2.3^	72.56 ^± 1.5^	44.33 ^± 5.8^	39.09 ^± 4.8^	35.88 ^± 4.0^	31.90 ^± 2.9^
BADGER	**71.55 ** ^±** **1.6^	**41.70 ** ^±** **6.5^	**36.70 ** ^±** **5.0^	**33.79 ** ^±** **4.0^	**30.40 ** ^±** **2.8^	**72.84 ** ^±** **1.4^	**45.70 ** ^±** **5.5^	**40.08 ** ^±** **4.3^	**36.77 ** ^±** **3.6^	**32.60 ** ^±** **2.8^

a Bold values indicate the best performance for each metric. BADGER consistently outperforms the model without PPAB across all metrics and split methods, demonstrating the effectiveness of the PPAB component. The superscript values represent the standard deviation.

### 3.4 Drug repurposing scenario

Drug repurposing involves identifying novel applications for existing approved drugs that exhibit chemical perturbation patterns in a target cell line similar to those of a reference drug. To demonstrate BADGER’s applicability in drug repurposing scenario, we perform a case study by first selecting a pair of approved drug and its targeted cell line that exists in our L1000 dataset. The selected drug is “sunitinib-malate,” which is recognized for targeting gastrointestinal stromal tumors, pancreatic cancer, and renal cell carcinoma while cell line is pancreatic cancer cell line, known as *YAPC* (Le Tourneau *et al.* 2007, Raymond *et al.* 2011).

The DrugBank is a publicly released database containing comprehensive information of approved drugs (Wishart *et al.* 2008). We conduct systematic screening of a large number of compounds contained in DrugBank and identify potential drug candidates that might not have been discovered through traditional target-based approaches. We first run inference on BADGER by feeding it the 2766 DrugBank approved drugs paired with the same *YAPC* cell embedding as input to obtain their attention weights extracted from the “PPAB” and predicted ranking of DEGs. As the proposed attention mechanism in BADGER’s “PPAB” provides insights in analyzing drug–pathway association patterns of unseen molecules, we exploit their computed attention matrices to prioritize which drug to be repurposed for effectively targeting *YAPC*.

Our study focuses on repositioning non-cancerous treatments toward anti-cancer applications. Exclusion of traditional anti-cancer treatments from current analysis rooted in the recognition that many cancer drugs are characterized by multi-target mechanism of action. To summarize, the goal of BADGER is to efficiently identify and utilize existing pharmacological agents in new therapeutic contexts.

Among the 2766 results, we selected potential drug candidates for repurposing based on the following criteria,

- Similarity of highly attended pathways based on the attention weights.
- Similarity of highly attended fragments based on the attention weights.
- Similarity of top 200 up-regulated genes.
- Similarity of top 200 down-regulated genes.
- Non-cancerous treatment drugs

**Figure 4.**
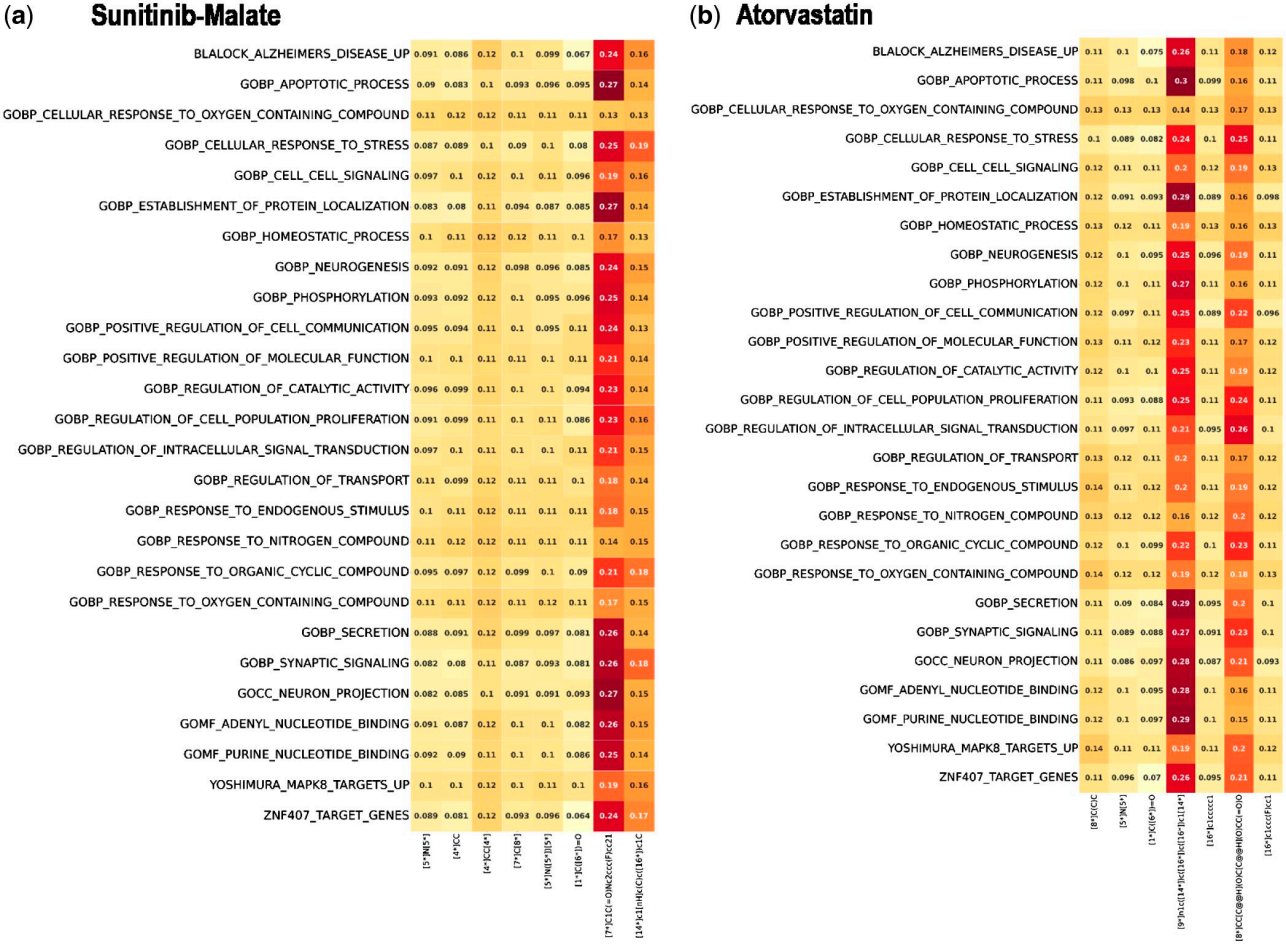
Comparative attention map analysis for pancreatic cancer treatment. This visualization represents the results from a Perturbation-Pathway cross attention block, using the YAPC pancreatic cancer cell line. It compares two drugs: (a) Sunitinib-malate, established treatment for pancreatic cancer, and (b) Atorvastatin, a potential repurposing candidate originally developed for cholesterol management and cardiovascular disease prevention. The *x*-axis of the map indicates different drug fragments, while *y*-axis denotes various biological pathways. The scores depict the extent of correlation between each drug fragment and the biological pathways. The attention map showcases a striking similarity in the highly attended pathways between Sunitinib-malate (a) and Atorvastatin (b) in the context of pancreatic cancer treatment, highlighting a 95.24% overlap. This significant congruence, analyzed using the YAPC pancreatic cancer cell line, underscores the potential repurposing of Atorvastatin, alongside the established efficacy of Sunitinib-malate.

Table 4 shows the 10 most promising drugs, which are “Silodosin,” “Atorvastatin,” “Vorapaxar,” “Manidipine,” “Bazedoxifene,” “Netarsudil,” “Avacopan,” “Elagolix,” “Vazegepant,” and “Cerivastatin.” To illustrate BADGER’s explainability being exploited in this process, we visualized the attention weights in heatmap comparing Sunitinib-malate with Atorvastatin as shown in Fig. 4. Sunitinib-malate, an established therapeutic for pancreatic cancer, and Atorvastatin, a drug primarily utilized for cholesterol regulation and cardiovascular disease prevention, is considered for potential repurposing in pancreatic cancer treatment.

**Table 4.** Evaluation of the similarity score for sunitinib-malate, a drug targeting pancreatic cancer, against the top 10 repurposing candidates.^a^

Rank	Drug	Attention-based Similarity Score		DGE-based Similarity Score		Average	Evidence
		Pathway	Fragment	Up-regulated	Down-regulated		
1	**Silodosin**	0.9091	0.5496	0.4184	0.8458	0.6807	–
**2**	**Atorvastatin**	0.9524	0.2447	0.5810	0.8884	0.6666	Cai *et al.* (2020)
**3**	**Vorapaxar**	1.0000	0.0920	0.6064	0.9092	0.6519	Schweickert *et al.* (2021)
**4**	**Manidipine**	1.0000	0.1201	0.5810	0.8953	0.6492	–
**5**	**Bazedoxifene**	0.9524	0.1553	0.5784	0.9045	0.6467	Burkhardt *et al.* (2019)
**6**	**Netarsudil**	1.0000	0.1855	0.5326	0.8657	0.6459	Barcelo *et al.* (2023)
**7**	**Avacopan**	0.9524	0.2082	0.5564	0.8657	0.6457	–
**8**	**Elagolix**	0.8000	0.1725	0.6736	0.9234	0.6424	Suo *et al.* (2019)
**9**	**Vazegepant**	0.9524	0.1328	0.5936	0.8657	0.6361	–
**10**	**Cerivastatin**	0.9524	0.0408	0.6194	0.9115	0.6310	Hao *et al.* (2019)

a The selection criteria for drugs similar to sunitinib-malate are based on four factors: (i) pathways significantly highlighted in the perturbation-pathway cross attention block, (ii) fragments receiving high attention in the same block, (iii) the top 200 up-regulated genes, and (iv) the top 200 down-regulated genes. We ranked the candidates based on the average score across these four criteria. “Evidence” refers to research that shows a direct or indirect connection between the candidate drug and pancreatic cancer.

Figure 4 presents a comparative attention map analysis examining drug–pathway relationships in pancreatic cancer treatment. The visualization, derived from a Perturbation-Pathway cross attention block using the YAPC pancreatic cancer cell line, compares Sunitinib-malate (an established pancreatic cancer treatment) with Atorvastatin (a cholesterol management drug). The attention heatmap illustrates the relationship between perturbations and pathways, examining not only DEG (differential expression levels) but also the biological activities of drugs at the pathway level. For Sunitinib-malate and Atorvastatin, when selecting attention weights above a certain threshold across 140 pathways, the heatmap reveals attention patterns to specific pathways. Notably, when comparing the pathways with high attention weights for both Sunitinib-malate and Atorvastatin, there is ∼95.24% overlap, indicating that these two drugs are associated with similar pathways when examining their effects at the pathway level.

The molecular analysis reveals distinct structural patterns in the highly attended fragments between the two compounds:

- Sunitinib-Malate
  - [14*]c1[nH]c(C)c([16*])c1C
  - [7*]C1C(=O)Nc2ccc(F)cc21
- Atorvastatin
  - [9*]n1c([14*])c([16*])c([16*])c1[14*]
  - [8*]CCC@@HCC@@HCC(=O)O

Despite the structural dissimilarity of these highly attended fragments, the pathway-level attention patterns, DEG similarity, and supporting literature indicate functional similarities between these compounds. This finding suggests potential opportunities for novel drug design approaches, particularly through the modification of residues based on Atorvastatin’s highly attended scaffolds. This analysis demonstrates BADGER’s ability to identify meaningful relationships between drugs at both the molecular and pathway levels, even when structural similarities are not immediately apparent, providing valuable insights for drug design and development.

We conduct a literature survey to establish the linkage between the top 10 candidate drugs and pancreatic cancer, categorizing the findings into three scenarios: (i) studies showing the drug’s effectiveness in inhibiting pancreatic cancer, (ii) research indicating the drug’s target protein aids in suppressing pancreatic cancer, and (iii) cases where no studies have been conducted. The following drugs have been identified with direct evidence of their impact on pancreatic cancer, while details about drugs of second case are provided in Supplementary Data S3.

#### 3.4.1 Atorvastatin

Cai *et al.* explores the potential of atorvastatin, a commonly used for lowering cholesterol, as an anti-cancer agent against pancreatic cancer, known for its aggressive nature and poor prognosis. Atorvastatin showed a significant reduction in cell proliferation, migration, and invasion in pancreatic cancer cell lines. The findings indicate that atorvastatin can inhibit the growth and spread of PC cells, potentially through the suppression of the neurotrophin signaling pathway. This suggests a new therapeutic application of atorvastatin in treating pancreatic cancer.

#### 3.4.2 Bazedoxifene

Burkhardt *et al.* (2019) investigates the potential of bazedoxifene, a drug primarily used for osteoporosis, as a treatment for advanced pancreatic and gastric tumors. Bazedoxifene has been identified as an effective inhibitor of the IL6/GP130/STAT3 pathway, which is implicated in the tumorigenesis and progression of these cancers. This suggests a new therapeutic option for treating pancreatic cancer.

#### 3.4.3 Cerivastatin

Hao *et al.* (2019) demonstrates that cerivastatin plays a significant role in pancreatic cancer by modulating the localization and activity of YAP, inhibiting important gene expressions related to cancer progression, and impacting cancer cell proliferation. These findings indicate that cerivastatin, a lipophilic statin, could have therapeutic potential in the treatment or prevention of pancreatic cancer.

The detailed results are provided in Supplementary Table S3. The selection of the top 10 drug candidates followed an approach. First, all compounds were sorted based on their “Average” score in descending order. To distinguish between anti-cancer and non-cancer drugs, we use two methods: (i) drugs with names ending in “ib” were automatically classified as anti-cancer drugs, as this suffix is conventionally used for protein kinase inhibitors in cancer therapy, and (ii) for all other drugs, we search DrugBank database to determine their primary therapeutic use and classified them accordingly. From this sorted and classified list, we select the top 10 scoring non-cancer drugs for further investigation. Due to space limitations in Supplementary Table S3, we include only the drugs up to the top 10 non-cancer candidates in our ranking.

This observation highlights the potential for wider applications of drug repositioning strategies, especially concerning “Silodosin,” “Manidipine,” “Avacopan,” and “Vazegepant.” While these four drugs have not been thoroughly researched for their effects against pancreatic cancer, existing literature on six other drugs points to a promising direction for further research. Building upon this, it becomes crucial to delve into the underlying mechanisms to clearly establish the connection between these drugs and their possible new therapeutic applications in treating pancreatic cancer.

## 4 Discussion

### 4.1 Generalized prediction capabilities of BADGER

BADGER utilizes prior knowledge of drug targets through pathway analysis to predict the effects of novel drugs based on their interactions with known pathways. By integrating comprehensive pathway data, BADGER extends its predictive capabilities to drugs that were not part of the training dataset. To address novel cell lines, BADGER employs similarity-based embeddings. These embeddings exploit the biological similarities between new and previously studied cell lines, enabling the model to generalize to unseen cellular contexts. This design allows for accurate predictions even with new cancer cell lines. The embeddings for new cell lines can be generated as 114-dimensional vectors by calculating similarity scores between the new cell line and the 114 anchor cell lines based on their control condition gene expression profiles. This design choice enables BADGER to generalize beyond the original cell lines by leveraging the patterns learned from the training data.

BADGER has shown strong performance on datasets with unseen cell lines and novel drug–cell pairs, validating its ability to make accurate predictions when both the drug and cell line are new. This generalizability is essential for practical applications in drug discovery and repurposing, where new cell types and drug compounds are frequently encountered. We conducted out-of-dataset tests to verify BADGER’s robustness by using the sci-Plex data. According to the external test results, BADGER demonstrates reliability and adaptability in handling diverse and previously untested data scenarios despite it having minimal performance differences with the baselines.

The success of drug repurposing strategies depends on the ability to offer treatment solutions for new, intractable diseases by leveraging similarities with existing diseases. This approach enables the swift application of known drugs to related but untested medical conditions, thereby accelerating the identification of potential therapies. In this context, the ability to make robust predictions for unseen cell lines is particularly crucial. BADGER’s proficiency in predicting responses in novel cell lines enhances the feasibility of drug repurposing, as it ensures that the model can generalize across various biological settings. This capability is essential for extending the utility of existing drugs to a broader range of diseases, thereby maximizing the impact of drug repurposing strategies. Emphasizing the importance of robust predictive capabilities for unseen cell lines highlights BADGER’s contribution to advancing the field of drug repurposing and expanding the therapeutic possibilities available for complex and emerging diseases.

### 4.2 Limitations and future work

Our study has yielded promising results, while also revealing several important limitations and areas for future improvement. The high dimensionality of genomic data, coupled with biological noise, presents one of the primary challenges in drug response prediction. This complexity is further compounded by the heterogeneity of biological data, making it difficult to achieve consistent performance across different datasets. When comparing our model’s top-K predictions against the ground truth’s top-200 DEGs among 978 genes, BADGER achieved 35–45% precision on our main dataset, with lower performance on the external dataset. While these results show improvement over existing approaches like DeepCE and CIGER, they also indicate substantial room for enhancement in future research. Our cross-validation analysis revealed relatively high standard deviations across the five folds, exceeding 5% in some cases. However, we found that baseline studies working with complex biological systems report comparable levels of variability, suggesting these fluctuations reflect inherent characteristics of the data rather than model limitations.

Second, we acknowledge a limitation in terms of the scope of the newly constructed L1000-based dataset. The L1000 dataset has internal noise, which stems from various sources including experimental variability, measurement errors, and biological variability. To enhance the dataset’s quality and reliability, we implemented a filtering process to mitigate noise impact by selecting data with chemical perturbations at 10M dosage and 24-h exposure time, which resulted in the removal of 84.15% of the data. While this filtering approach effectively improves data integrity, it also means excluding potentially valuable data points, thus limiting the dataset’s comprehensiveness. In particular, our current model cannot handle samples with different dosages of the same drug, as the training data only includes a single standardized concentration. Extending the model to incorporate dose-dependent effects remains an important direction for future work.

Moreover, the training dataset used in the study represents a subset of the possible universe of small molecules and cell lines. The dataset includes 6737 small molecules and 114 cell lines, which, despite being substantial, is limited compared to the full range of existing compounds and cell types relevant to drug discovery and biological research. The constrained dataset size might impact the generalizability of the study’s findings. Models trained on limited data may not capture the full spectrum of biological variability and compound efficacy. To further enhance the robustness and applicability of the model, future studies could consider expanding the dataset, incorporating a wider array of compounds and cell types. Additionally, integrating data from complementary sources or employing techniques that enhance data augmentation and diversity could also help in mitigating the impact of the dataset’s limited scope.

Lastly, we speculate that one of our limitations lies in our drug representation method. The current approach, involving BRICS decomposition and Morgan Fingerprints, may not fully capture the chemical characteristics of unseen molecules. This limitation could be contributing to the model’s performance ceiling and its challenges with generalizability, as evidenced by the lower performance on the external dataset.

Based on these findings and limitations, we propose several interconnected directions for future research. Primarily, we plan to enhance our dataset by integrating complementary sources and employing data augmentation techniques, which will improve its comprehensiveness and quality. This improved dataset will then enable us to seek better alternatives for drug representation, aiming to characterize molecules not only with structure-related properties but also with biologically relevant features. With this enhanced data and representation, we will be better positioned to explore strategies for reducing false positives, potentially by incorporating additional biological knowledge or developing more sophisticated feature selection methods. These improvements, in turn, will contribute to our key focus of enhancing model robustness across diverse datasets, directly addressing the challenge of lower performance on external datasets.

## 5 Conclusion

In this article, we propose BADGER model for predicting the ranking of differential gene expression in response to chemical perturbations. The architecture of BADGER is distinctively structured, incorporating three stacked attention blocks, each tailored to capture different aspects of the intricate biological interactions. Collectively, these attention blocks enable BADGER to model the mechanisms underlying chemical perturbations. This reflects a deep understanding of biological systems, positioning it as a potentially powerful tool in computational biology and drug repurposing.

## Supplementary Material

vbaf029_Supplementary_Data

## Data Availability

All code and data used in this study are available at https://github.com/dmis-lab/BADGER.git.

## Appendix

### 1 Implementation details for the attention blocks in BADGER

The Perturbation-Pathway Cross-Attention Block mathematically notated as “PPAB” that takes a set of pertubation embeddings $X_{M+C}$ as input is mathematically expressed as follows:

(A.1) $$X_{M+C}^{+}=X_{M+C}\cup \left\{p_{F}\right\}$$

(A.2) $$X_{P}=\mathrm{PPAB}(X_{M+C}^{+})$$

(A.3) $$\mathrm{PPAB}(X_{M+C}^{+})=H+\mathrm{RFF}(\mathrm{SNorm}(H))$$

(A.4) $$H=\mathrm{MHA}(\mathrm{SNorm}(P),\mathrm{SNorm}(X_{M+C}^{+}),\mathrm{SNorm}(X_{M+C}^{+}))$$

where $X_{M+C}^{+}\in R^{(k+1)\times d}$ is a set of *k *+* *1 *d*-dimensional fragment-level perturbation embeddings including pseudo-fragment $p_{F},\;X_{P}\in R^{140\times d}$ is a set of 140 *d*-dimensional *perturbation-contextualized* pathway embeddings. *RFF* is row-wise feed forward layer, *SNorm* is Set-based Normalization (Zhang *et al.* 2022). *MHA* is Multihead Attention layer that takes its internal trainable pathway embeddings $P\in R^{140\times d}$ as query and $X_{M+C}^{+}$ as key-value. The pairwise attention scoring method is based on Addition Attention (Bahdanau *et al.* 2014) while the number of heads is two in this layer.

The Pathway-Gene Cross-Attention Block mathematically notated as “PGAB” that takes a set of chemical-induced pathway embeddings $X_{P}$ as input is mathematically expressed as follows:

(A.5) $$X_{G}=\mathrm{PGAB}(X_{P})\mathrm{PGAB}(X)=H+\mathrm{RFF}(\mathrm{SNorm}(H))$$

(A.6) $$H=\mathrm{MHA}(\mathrm{SNorm}(G),\mathrm{SNorm}(X_{P}),\mathrm{SNorm}(X_{P}))$$

where $X_{G}\in R^{978\times d}$ is a set of 978 *d*-dimensional updated landmark gene embeddings, *MHA* is Multihead Attention layer that takes its internal trainable gene embeddings $G\in R^{978\times d}$ as query input and $X_{P}$ as key-value input. The pairwise attention scoring method and number of attention heads is same as above.

The Gene–Gene Self-Attention Block mathematically notated as “GGAB” that takes a set of updated landmark gene embeddings $X_{T}$ as input is mathematically expressed as follows:

(A.7) $$X_{Z}=\mathrm{GGAB}(X_{G}^{*})$$

(A.8) GGAB(X)=H+RFF(SNorm(H))

(A.9) $$H=\mathrm{MHA}(\mathrm{SNorm}(X_{G}^{*}),\mathrm{SNorm}(X_{G}^{*}),\mathrm{SNorm}(X_{G}^{*}))$$

where $X_{Z}\in R^{978\times d}$ is a set of 978 *d*-dimensional final state landmark gene embeddings.

### 2 Gene ontology embedding construction

We utilized GO terms to create pathway embeddings for our model. GO terms provide a standardized vocabulary describing the biological processes, molecular functions, and cellular components associated with genes, offering a comprehensive framework for capturing gene–pathway relationships. To construct the embeddings, we first collected a comprehensive set of GO terms related to our genes of interest. For each gene, we created a binary vector where the vector length equals the total number of considered GO terms, with each position corresponding to a specific GO term. In this vector, we assign a value of 1 if the gene is associated with a particular GO term, and 0 if it is not. This process resulted in a sparse binary matrix where rows represent individual genes and columns represent GO terms, with matrix entries (1 or 0) indicating the presence or absence of gene–term associations. The resulting one-hot encoded representation serves as our initial GO embedding, which is subsequently utilized in the Pathway-Gene Cross-Attention block. This approach enables the incorporation of valuable biological knowledge into our model, allowing it to leverage the functional relationships between genes and their associated biological pathways during the attention computation process.
