# Supplementary material for: BADGER: biologically-aware interpretable differential gene expression ranking model
Source: Bioinform Adv. 2025 Feb 18;5(1):vbaf029. doi: 10.1093/bioadv/vbaf029 (PMC11978390; doi:10.1093/bioadv/vbaf029)
Supplement: vbaf029_Supplementary_Data [file vbaf029_supplementary_data.zip › BADGER_supplementary_final.pdf]

Bioinformatics Advances, 2024, pp. 1–15

doi: DOI HERE
Advance Access Publication Date: Day Month Year
Paper

PAPER

Supplement to BADGER: Biologically-Aware Interpretable Differential Gene Expression Ranking Model

Hajung Kim,[1,†] Mogan Gim,[2,†,‡] Seungheun Baek,[1] Soyon Park,[1]
Sunkyu Kim[3,*] and Jaewoo Kang[1,3,*]

[1]Department of Computer Science and Engineering, Korea University, Seoul, 02841, Korea, [2]Department of Biomedical Engineering, Hankuk University of Foreign Studies, 17035, Korea and [3]AIGEN Sciences

[†]The authors wish it to be known that the first two authors contributed equally.

[*]Corresponding author. Engineering, Korea University, Seoul, 02841, Korea

Email: kangj@korea.ac.kr[‡]This work was done while the author was a postdoctoral researcher at Korea University

FOR PUBLISHER ONLY Received on Date Month Year; revised on Date Month Year; accepted on Date Month Year

Abstract

Motivation Understanding which genes are significantly affected by drugs is crucial for drug repurposing, as drugs targeting specific pathways in one disease might be effective in another with similar genetic profiles. By analyzing gene expression changes in cells before and after drug treatment, we can identify the genes most impacted by drugs.

Results The Biologically-Aware Interpretable Differential Gene Expression Ranking (BADGER) model is an interpretable model designed to predict gene expression changes resulting from interactions between cancer cell lines and chemical compounds. The model enhances explainability through integration of prior knowledge about drug targets via pathway information, handles novel cancer cell lines through similarity-based embedding, and employs three attention blocks that mimic the cascading effects of chemical compounds. This model overcomes previous limitations of cell line range and explainability constraints in drug-cell response studies. The model demonstrates superior performance over baselines in both unseen cell and unseen pair split evaluations, showing robust prediction capabilities for untested drug-cell line combinations.

Availability and implementation This makes it particularly valuable for drug repurposing scenarios, especially in developing therapeutic plans for new or resistant diseases by leveraging similarities with other diseases. All code and data used in this study are available at https://github.com/dmis-lab/BADGER.git.

Key words: Differential Gene Expression, Drug repurposing, Deep Learning Model

© The Author 2024. Published by Oxford University Press. All rights reserved. For permissions, please e-mail: journals.permissions@oup.com

S1. Correlation Analysis of Expression Predictions

To provide a comprehensive evaluation of model performance, we analyze both Pearson and Spearman correlation coefficients across different data split settings. The correlation analysis reveals varying degrees of predictive capability across different experimental contexts. In the perturbation split setting, CIGER achieved the highest correlations (Pearson: 0.287, Spearman: 0.250), while DeepCE performed best in the cell split setting (Pearson: 0.292, Spearman: 0.275). BADGER demonstrated superior performance in the pair split setting (Pearson: 0.304, Spearman: 0.292). The relatively low correlation values (Pearson and Spearman correlations ranging from 0.2 to 0.3) indicate that the models' ability to predict overall gene expression changes is limited. However, as shown in our main analysis, the models demonstrate better performance in identifying genes with substantial expression changes, suggesting their utility may be more specific to detecting major transcriptional responses rather than capturing genome-wide expression patterns.

**Model**	**Pert Split**		**Cell Split**		**Pair Split**	
	Pearson	Spearman	Pearson	Spearman	Pearson	Spearman
AverageRank	0.2199	0.2050	0.1955	0.1884	0.2015	0.1943
kNN	0.1955	0.1748	0.2354	0.2173	0.1464	0.1332
MLP	0.1299	0.2099	0.1141	0.1919	0.0463	0.1556
DeepCE	0.2283	0.1969	**0.2919**	**0.2752**	0.1850	0.1746
CIGER	**0.2869**	**0.2504**	0.1938	0.1814	0.2731	0.2536
BADGER	0.2489	0.2245	0.2409	0.2145	**0.3036**	**0.2921**

Table S1. Correlation coefficients across different split settings for gene expression prediction models

S2. Model Architecture Details and Training Convergence

The parameter counts and memory requirements for each model architecture are summarized. BADGER achieves its performance with a relatively compact architecture (852,628 total parameters, 657,027 trainable), requiring only 3.33 MB of memory. In comparison, while MLP has the largest architecture with approximately 4.26M parameters, DeepCE maintains a similar parameter count to BADGER (808,473 total parameters), and CIGER falls in between with 1.45M parameters. Notably, BADGER and DeepCE incorporate non-trainable parameters as part of their architecture design, while MLP and CIGER utilize fully trainable parameters. The training dynamics are monitored using an early stopping mechanism with a patience of 10 epochs, evaluated on validation loss. The convergence patterns varied across different cross-validation folds, with models reaching optimal performance between epochs 12 and 31. This variation in convergence timing suggests that different aspects of the perturbation response patterns required varying amounts of training to be effectively captured by the models.

**Model**	**Total Params**	**Trainable**	**Non-trainable**	**Size (KB)**
DeepCE	808,473	683,289	125,184	3,158.10
CIGER	1,445,079	1,445,079	0	5,644.84
BADGER	852,628	657,027	195,601	3,330.58

Table S2. Comparison of model architectures showing total number of parameters, trainable and non-trainable parameters, and model sizes. BADGER achieves its functionality with a relatively compact architecture (852K parameters), while maintaining efficiency through selective parameter freezing (195K non-trainable parameters).

S3. Repurposing candidate drugs

We conducted a comprehensive literature review to establish the connections between the top 10 candidate drugs and pancreatic cancer. Our findings are categorized into three scenarios: 1.Studies demonstrating the drug's effectiveness in inhibiting pancreatic cancer. 2. Research indicating that the drug's target protein plays a crucial role in suppressing pancreatic cancer. 3. Cases where no relevant studies have been conducted. Following drugs are categorized into second scenario. They are associated with specific targets, and existing literature demonstrates that inhibiting these target proteins significantly contributes to inhibiting cancer proliferation.

Vorapaxar

Vorapaxar functions as a thrombin receptor antagonist by reversibly inhibiting the protease-activated receptor-1 (PAR-1). Schweickert et al. (2021) suggests that siliencing or inhibiting the PAR-1 in pancreatic ductal adenocarcinoma cells can hinder the growth of pnacreatic cancer. The PAR-1 plays a significant role in pancreatic cancer growth by promoting immune evasion, and thus targeting PAR1 could be potential strategy for inhibiting the growth of pancreatic cancer.

Netarsudil

Netarsudil, commonly used for treating glaucoma and ocular hypertension, is categorized as a rho-kinase inhibitor, targeting rho-associated protein kinases 1 and 2 (ROCK1 and ROCK2). Barcelo et al. (2023) have suggested the potential of ROCK inhibitor to hinder invasion and metastasis. Specifically in pancreatic cancer, early intervention with a ROCK inhibitor can

diminish the likelihood of cancer cell extravasation and metastasis. This highlights the potential of Netarsudil as a treatment option for early-stage pancreatic cancer.

Elagolix

Suo et al. (2019) suggests that the regulation of Gonadotropin-releasing hormone (GnRH) expression plays a crucial role in the development of pancreatic cancer, indicating that it could be a vailable target for treating patients with this disease. Elagolix, a GnRH receptor antagonist, has potential in this context, underscoring its therapeutic promise for pancreatic cancer management.

Drug Candidate	Attention-based Similarity Score		DGE-based Similarity Score		Average
	Pathway	Fragment	Up-regulated	Down-regulated	
Sunitinib (reference)	1	1	1	1	1
Vorolanib	1	1	0.73913	0.930521	0.91741275
Amcasertib	0.833333	1	0.801802	0.947434	0.89564225
SU-11652	1	1	0.659751	0.916256	0.89400175
Nintedanib	0.909091	0.774818	0.646091	0.883777	0.80344425
Fingolimod	0.636364		0.646091	0.913899	0.732118
Esaxerenone	0.95	0.553191	0.498127	0.890644	0.7229905
(11R)-10-acetyl-11-(2,4-dichlorophenyl)-6-hydroxy-3,3-dimethyl-2,3,4,5,10,11-hexahydro-1H-dibenzo[b,e][1,4]diazepin-1-one	0.590909		0.646091	0.906863	0.714621
AZD-4547	1	0.111111	0.777778	0.954774	0.71091575
Dubermatinib	0.952381	0.140741	0.785714	0.962169	0.71025125
Efonidipine	0.952381	0.170845	0.769912	0.945	0.7095345
Flumatinib	0.909091	0.403361	0.612903	0.897561	0.705729
Evacetrapib	0.952381	0.337644	0.587302	0.932919	0.7025615
Osimertinib	0.909091	0.101399	0.826484	0.96713	0.701026
(S)-wiskostatin	0.636364		0.568627	0.883777	0.696256
Rociletinib	0.909091	0.153153	0.777778	0.94015	0.695043
HM-43239	0.952381	0.140741	0.724138	0.957233	0.69362325
(3R)-3-cyclopentyl-7-[(4-methylpiperazin-1-yl)sulfonyl]-3,4-dihydro-2H-1,2-benzothiazine 1,1-dioxide	0.636364		0.544402	0.897561	0.6927756667
Ceritinib	0.863636	0.176715	0.777778	0.952321	0.6926125
LY-2456302	0.952381	0.185484	0.709402	0.920988	0.69206375
Gandotinib	0.952381	0.123596	0.73913	0.937733	0.68821
BMS-929075	0.952381	0.146712	0.694915	0.942572	0.684145
Tesevatinib	0.952381	0.066798	0.754386	0.952321	0.6814715
Mibefradil	0.909091	0.266983	0.632653	0.916256	0.68124575
Silodosin	0.909091	0.549637	0.41844	0.845789	0.68073925
Ensartinib	1	0.122581	0.67364	0.911548	0.67694225
Poziotinib	0.952381	0.122699	0.702128	0.930521	0.67693225
VS-4718	0.909091	0.201923	0.652893	0.932919	0.6742065
ASP-3026	0.952381	0.037736	0.746725	0.959698	0.674135
Spebrutinib	1	0.150376	0.632653	0.906863	0.672473
Bosutinib	0.952381	0.02968	0.769912	0.935323	0.671824
Fluazuron	1	0.169014	0.606426	0.911548	0.671747
ALK-4290	0.952381	0.403361	0.509434	0.819883	0.67126475
(3Z)-6-(4-HYDROXY-3-METHOXYPHENYL)-3-(1H-PYRROL-2-YLMETHYLENE)-1,3-DIHYDRO-2H-INDOL-2-ONE	0.681818	0.774818	0.388889	0.839243	0.671192
Indole Naphthyridinone	0.863636	0.255259	0.659751	0.9022	0.6702115
CEP-37440	0.909091	0.165339	0.687764	0.918619	0.67020325
Rilapladib	0.769231	0.103226	0.843318	0.964646	0.67010525
4-{[5-chloro-4-(1H-indol-3-yl)pyrimidin-2-yl]amino}-N-ethylpiperidine-1-carboxamide	0.818182	0.140741	0.754386	0.954774	0.66702075
Atorvastatin	0.952381	0.244681	0.581028	0.88835	0.66661
Capromorelin	1	0.14157	0.626016	0.897561	0.66628675
(S)-N-(1-(3-CHLORO-4-FLUOROPHENYL)-2-HYDROXYETHYL)-4-(4-(3-CHLOROPHENYL)-1H-PYRAZOL-3-YL)-1H-PYRROLE-2-CARBOXAMIDE	0.952381	0.244681	0.55642	0.909202	0.665671
AZD-5423	1	0.085837	0.659751	0.911548	0.664284
Cediranib	0.863636	0.163717	0.702128	0.925743	0.663806
PF-114	0.95	0.174118	0.619433	0.911548	0.66377475
Flufenoxuron	1	0.093897	0.639344	0.918619	0.662965
Surufatinib	1	0.205882	0.568627	0.874699	0.662302
AEE-788	1	0.123016	0.632653	0.892944	0.66215325
(S)-N-(4-carbamimidoylbenzyl)-1-(2-(cyclopentyloxy)ethanoyl)pyrrolidine-2-carboxamide	1	0.132184	0.6	0.916256	0.66211
CPI-1205	0.909091	0.26087	0.593625	0.879227	0.66070325
Ribociclib	0.952381	0.123016	0.646091	0.920988	0.660619
Abivertinib	0.952381	0.123016	0.646091	0.918619	0.66002675
Darapladib	0.833333	0.14479	0.724138	0.932919	0.658795
(Z)-2-[2-(4-methylpiperazin-1-yl)benzyl]diazenecarbothioamide	0.636364		0.492537	0.845789	0.65823
JPC-3210	0.904762	0.179856	0.626016	0.920988	0.6579055
Satavaptan	0.909091	0.285714	0.5625	0.872443	0.657437
TAS-108	1	0.183439	0.568627	0.87696	0.6572565
X-396	1	0.122581	0.619433	0.886061	0.65701875
Encorafenib	0.909091	0.111111	0.687764	0.918619	0.65664625
Imatinib	1	0.159292	0.581028	0.883777	0.65602425
Ravoxertinib	0.818182	0.115044	0.746725	0.94015	0.65502525
Balanol Analog 2	1	0.056	0.646091	0.913899	0.6539975
AFN-1252	0.863636	0.255259	0.593625	0.9022	0.65368
Niguldipine	0.952381	0.120066	0.626016	0.913899	0.6530905
Dexniguldipine	0.952381	0.120066	0.626016	0.913899	0.6530905
(2R)-N-HYDROXY-2-[(3S)-3-METHYL-3-{4-[(2-METHYLQUINOLIN-4-YL)METHOXY]PHENYL}-2-OXOPYRROLIDIN-1-YL]PROPANAMIDE	0.863636	0.192593	0.646091	0.909202	0.6528805
Fedratinib	1	0.243902	0.486989	0.879227	0.6525295
Vandetanib	0.952381	0.106195	0.646091	0.904529	0.652299
GDC-0084	0.636364		0.465201	0.854589	0.6520513333
Vorapaxar	1	0.09201	0.606426	0.909202	0.6519095
Radotinib	0.952381	0.213115	0.544402	0.897561	0.65186475
Tegaserod	0.636364		0.476015	0.84142	0.6512663333
(3R)-3-cyclopentyl-6-methyl-7-[(4-methylpiperazin-1-yl)sulfonyl]-3,4-dihydro-2H-1,2-benzothiazine 1,1-dioxide	0.636364		0.481481	0.834906	0.650917
Tiracizine	0.95	0.264184	0.538462	0.850178	0.650706
Agerafenib	1	0.089431	0.632653	0.879227	0.65032775
PF-06459988	0.952381	0.104956	0.612903	0.930521	0.65019025
3-[5-(1H-IMIDAZOL-1-YL)-7-METHYL-1H-BENZIMIDAZOL-2-YL]-4-[(PYRIDIN-2-YLMETHYL)AMINO]PYRIDIN-2(1H)-ONE	0.863636	0.142857	0.652893	0.937733	0.64927975
Manidipine	1	0.120066	0.581028	0.89525	0.649086
4-[(1S,2S,5S,9R)-5-(HYDROXYMETHYL)-8,9-DIMETHYL-3-OXABICYCLO[3.3.1]NON-7-EN-2-YL]PHENOL	0.590909		0.503759	0.852381	0.6490163333
6-Chloro-2-(2-Hydroxy-Biphenyl-3-Yl)-1h-Indole-5-Carboxamidine	0.590909		0.492537	0.863473	0.648973
Dasatinib	0.952381	0.188976	0.538462	0.913899	0.6484295
4-[3-(4-CHLOROPHENYL)-1H-PYRAZOL-5-YL]PIPERIDINE	0.590909		0.465201	0.88835	0.6481533333
5-[(Z)-(5-Chloro-2-oxo-1,2-dihydro-3H-indol-3-ylidene)methyl]-N,2,4-trimethyl-1H-pyrrole-3-carboxamide	0.681818	0.655936	0.384083	0.870192	0.64800725
Cabozantinib	1	0.115207	0.5625	0.911548	0.64731375
N-(1-ISOPROPYLPIPERIDIN-4-YL)-1-(3-METHOXYBENZYL)-1H-INDOLE-2-CARBOXAMIDE	1	0.127358	0.568627	0.892944	0.64723225
Pyrotinib	0.869565	0.149701	0.652893	0.916256	0.64710375
Saredutant	1	0.020642	0.652893	0.913899	0.6468585
Pipendoxifene	0.952381	0.155263	0.574803	0.904529	0.646744
Bazedoxifene	0.952381	0.155263	0.574803	0.904529	0.646744
4-[(4-METHYL-1-PIPERAZINYL)METHYL]-N-[3-[[4-(3-PYRIDINYL)-2-PYRIMIDINYL]AMINO]PHENYL]-BENZAMIDE	1	0.06	0.626016	0.899878	0.6464735
(S)-N-(4-carbamimidoylbenzyl)-1-(2-(cyclohexyloxy)ethanoyl)pyrrolidine-2-carboxamide	1	0.132184	0.544402	0.909202	0.646447
[4-({4-[(5-cyclopropyl-1H-pyrazol-3-yl)amino]quinazolin-2-yl}amino)phenyl]acetonitrile	0.863636	0.092664	0.694915	0.932919	0.6460335
Netarsudil	1	0.185484	0.532567	0.865707	0.6459395
2-amino-4-[2,4-dichloro-5-(2-pyrrolidin-1-ylethoxy)phenyl]-N-ethylthieno[2,3-d]pyrimidine-6-carboxamide	0.952381	0.118694	0.593625	0.918619	0.64582975
Avacopan	0.952381	0.208232	0.55642	0.865707	0.645685
GSK-376501	0.909091	0.127358	0.619433	0.925743	0.64540625
GS-9256	0.833333	0.164076	0.659751	0.920988	0.644537
AZD-1656	0.909091	0.06	0.687764	0.920988	0.64446075
3-[(3-sec-butyl-4-hydroxybenzoyl)amino]azepan-4-yl 4-(2-hydroxy-5-methoxybenzoyl)benzoate	0.909091	0.024887	0.702128	0.94015	0.644064
Pelitinib	1	0.149701	0.538462	0.886061	0.643556
Pipradrol	0.590909		0.509434	0.828437	0.6429266667
Elagolix	0.8	0.172549	0.67364	0.923362	0.64238775
Canertinib	0.952381	0.019185	0.67364	0.923362	0.642142
Nilotinib	0.952381	0.213115	0.509434	0.892944	0.6419685
N-BENZYL-4-[4-(3-CHLOROPHENYL)-1H-PYRAZOL-3-YL]-1H-PYRROLE-2-CARBOXAMIDE	1	0.244681	0.41844	0.904529	0.6419125
Cilnidipine	1	0.120066	0.544402	0.899878	0.6410865
(2-{[(4-BROMO-2-FLUOROBENZYL)AMINO]CARBONYL}-5-CHLOROPHENOXY)ACETIC ACID	0.95	0.106195	0.619433	0.88835	0.6409945
Sapitinib	1	0.092664	0.574803	0.892944	0.64010275
Golvatinib	0.833333	0.02968	0.754386	0.942572	0.63999275
Implitapide	0.952381	0.157583	0.55642	0.890644	0.639257
Otenabant	0.95	0.088353	0.619433	0.897561	0.63883675
Itacitinib	0.952381	0.014184	0.666667	0.920988	0.638555
1-Tert-Butyl-3-(4-Chloro-Phenyl)-1h-Pyrazolo[3,4-D]Pyrimidin-4-Ylamine	0.590909		0.438849	0.883777	0.637845
Clofazimine	0.590909		0.428571	0.892944	0.6374746667
5-(2-ETHOXYETHYL)-5-[4-(4-FLUOROPHENOXY)PHENOXY]PYRIMIDINE-2,4,6(1H,3H,5H)-TRIONE	0.952381	0.07451	0.606426	0.916256	0.63739325
Elacestrant	0.909091	0.169643	0.593625	0.87696	0.63732975
3-(3,5-Dibromo-4-Hydroxy-Benzoyl)-2-Ethyl-Benzofuran-6-Sulfonic Acid [4-(Thiazol-2-Ylsulfamoyl)-Phenyl]-Amide	0.952381	0.108833	0.606426	0.881499	0.63728475
HKI-357	0.8	0.149701	0.680672	0.918619	0.637248
KX-01	0.909091	0.201923	0.544402	0.892944	0.63709
LY-377604	0.952381	0.10757	0.606426	0.881499	0.636969
Gefitinib	0.952381	0.115044	0.581028	0.897561	0.6365035
Vazegepant	0.952381	0.132787	0.593625	0.865707	0.636125
Ulixertinib	0.952381	0.244681	0.486989	0.85902	0.63576775
1-(2,6-Dichlorophenyl)-5-(2,4-Difluorophenyl)-7-Piperazin-1-Yl-3,4-Dihydroquinazolin-2(1h)-One	0.636364		0.444043	0.82415	0.6348523333
[4-({4-[(5-cyclopropyl-1H-pyrazol-3-yl)amino]-6-(methylamino)pyrimidin-2-yl}amino)phenyl]acetonitrile	0.952381	0.010101	0.646091	0.930521	0.6347735
1-{4-[4-Amino-6-(4-methoxyphenyl)furo[2,3-d]pyrimidin-5-yl]phenyl}-3-[2-fluoro-5-(trifluoromethyl)phenyl]urea	0.95	0.142259	0.550388	0.89525	0.63447425
Dacomitinib	0.95	0.115044	0.568627	0.9022	0.63396775
Velusetrag	0.952381	0.095563	0.581028	0.906863	0.63395875
Masitinib	1	0.159292	0.520913	0.852381	0.6331465
Brigatinib	0.952381	0.140741	0.538462	0.899878	0.6328655
1-(3-HYDROXYPROPYL)-2-[(3-NITROBENZOYL)AMINO]-1H-BENZIMIDAZOL-5-YL PIVALATE	1	0.021327	0.606426	0.9022	0.63248825
Cerivastatin	0.952381	0.040816	0.619433	0.911548	0.6310445

Table S3. Full list of drug repurposing candidates ranked by average similarity score. The highlight ones were selected as final candidates based on our criteria related to non-cancerous drugs.

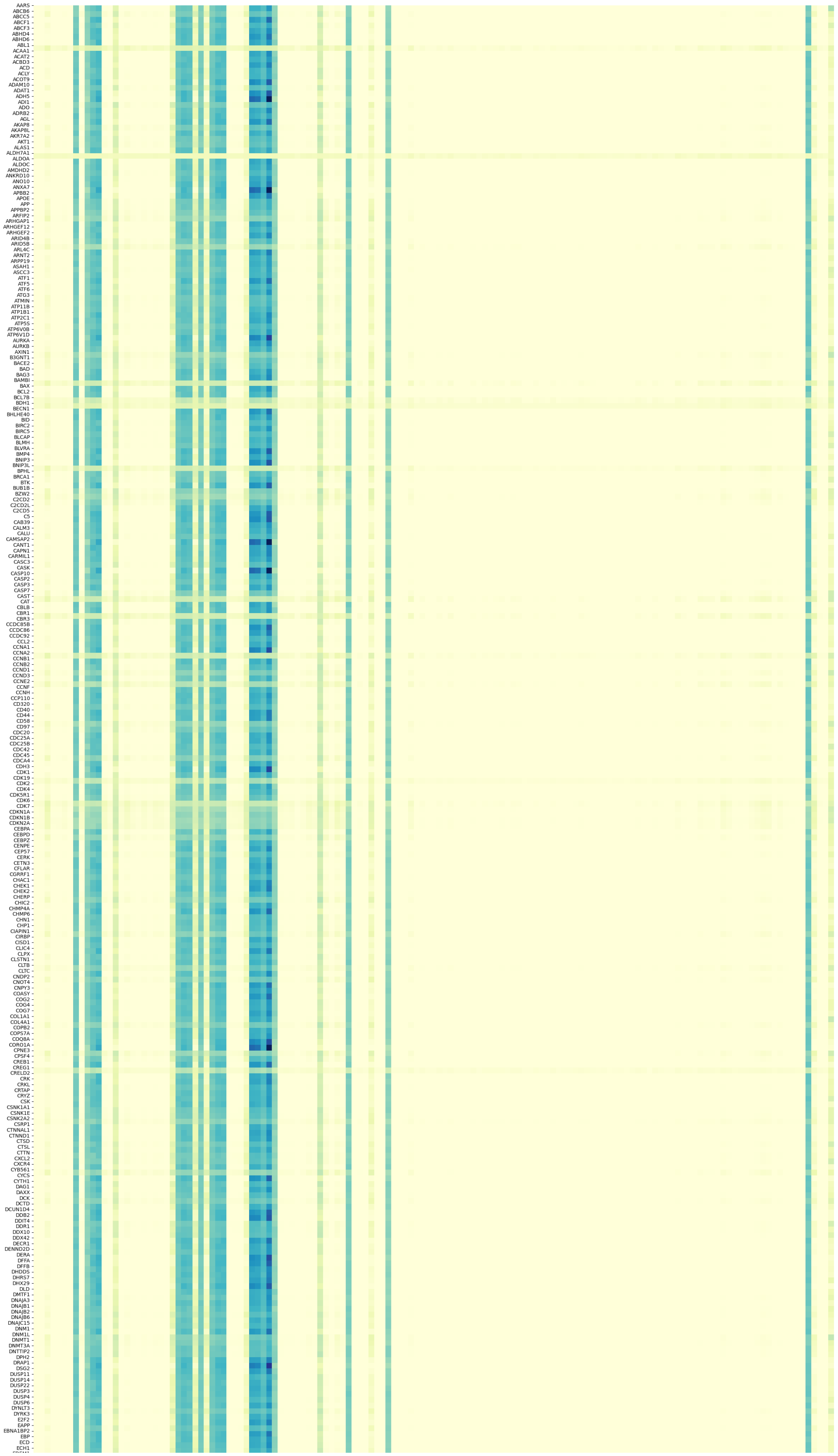

Fig. S1. Attention weights computed between the 978 shareable landmark gene embeddings and 140 perturbation-contextualized pathway embeddings, extracted from BADGER's Pathway-Gene Cross Attention Block when the input YAPC cell is treated with Sunitinib-Malate (1/4 figure).

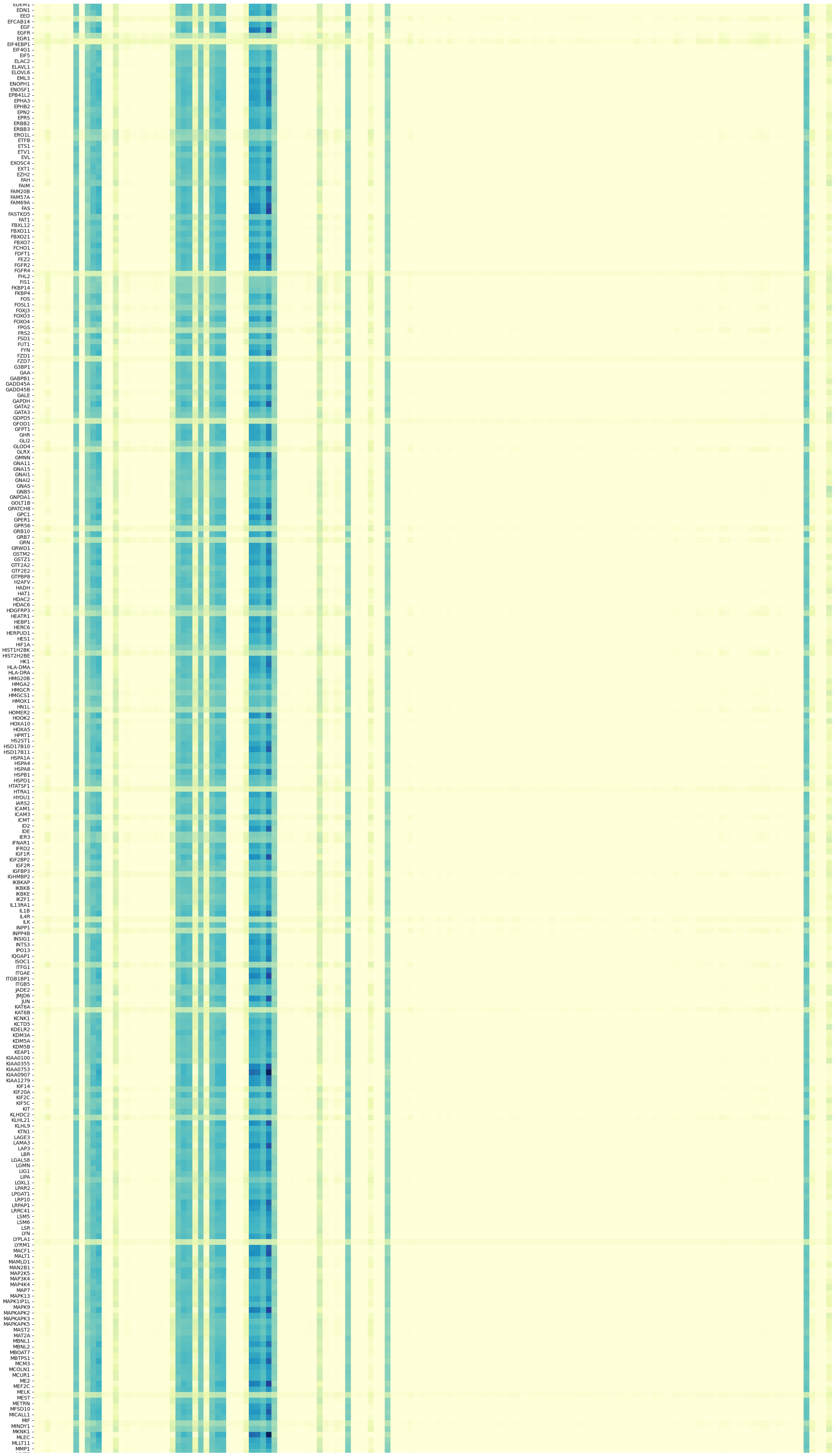

Fig. S2. Attention weights computed between the 978 shareable landmark gene embeddings and 140 perturbation-contextualized pathway embeddings, extracted from BADGER's Pathway-Gene Cross Attention Block when the input YAPC cell is treated with Sunitinib-Malate (2/4 figure).

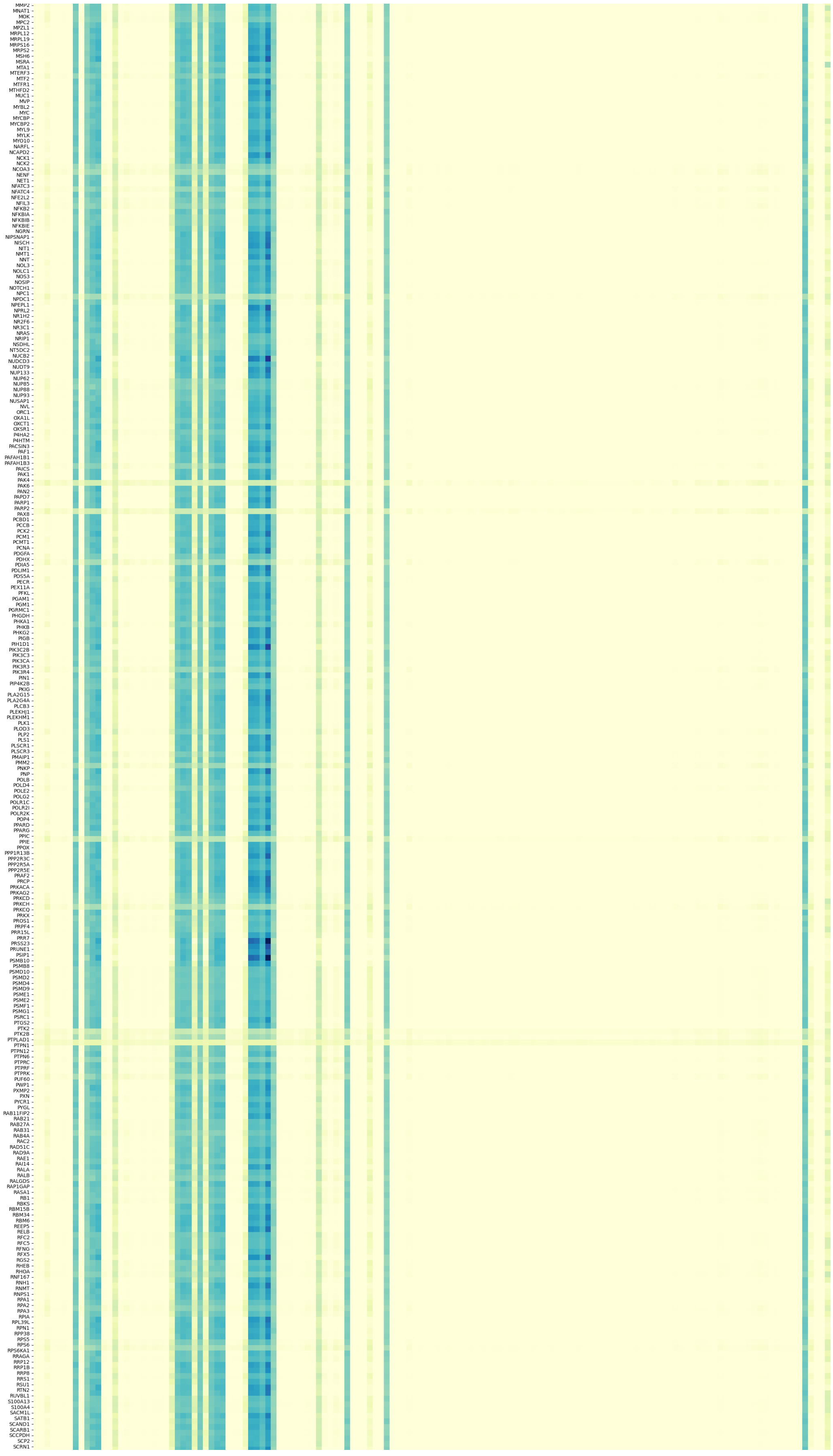

Fig. S3. Attention weights computed between the 978 shareable landmark gene embeddings and 140 perturbation-contextualized pathway embeddings, extracted from BADGER's Pathway-Gene Cross Attention Block when the input YAPC cell is treated with Sunitinib-Malate (3/4 figure).

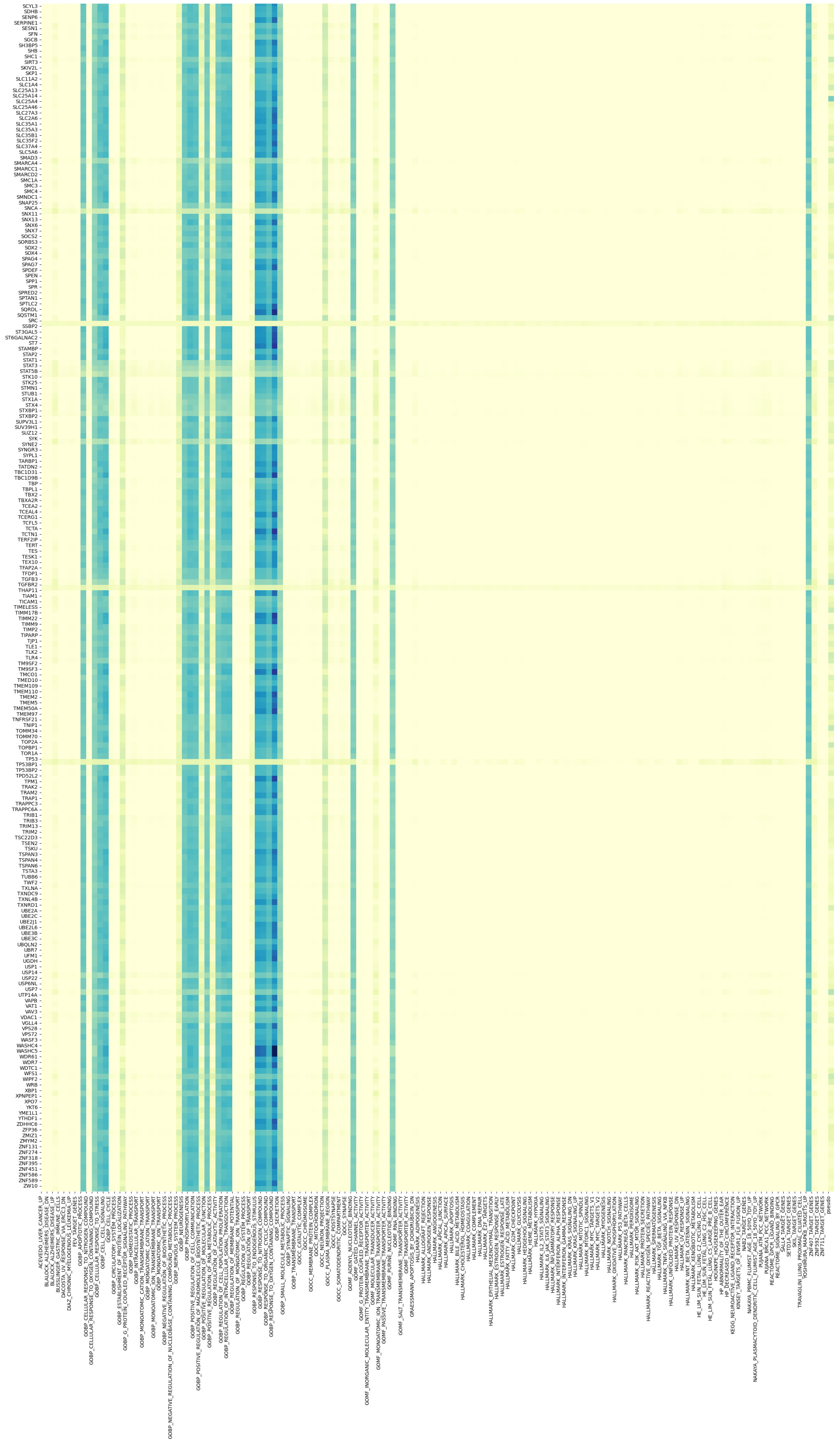

Fig. S4. Attention weights computed between the 978 shareable landmark gene embeddings and 140 perturbation-contextualized pathway embeddings, extracted from BADGER's Pathway-Gene Cross Attention Block when the input YAPC cell is treated with Sunitinib-Malate (4/4 figure).

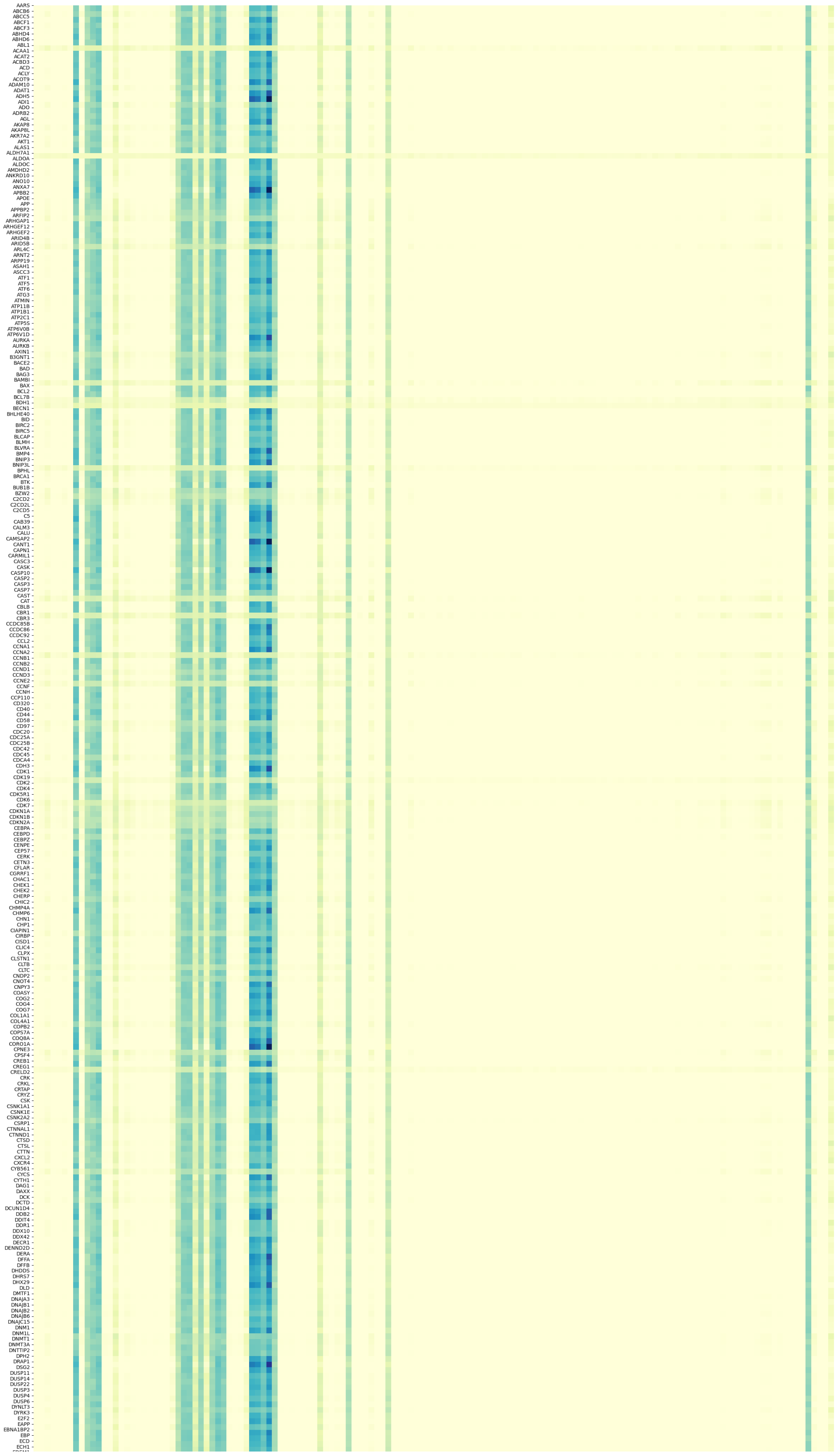

Fig. S5. Attention weights computed between the 978 shareable landmark gene embeddings and 140 perturbation-contextualized pathway embeddings, extracted from BADGER's Pathway-Gene Cross Attention Block when the input YAPC cell is treated with Atorvastatin (1/4 figure).

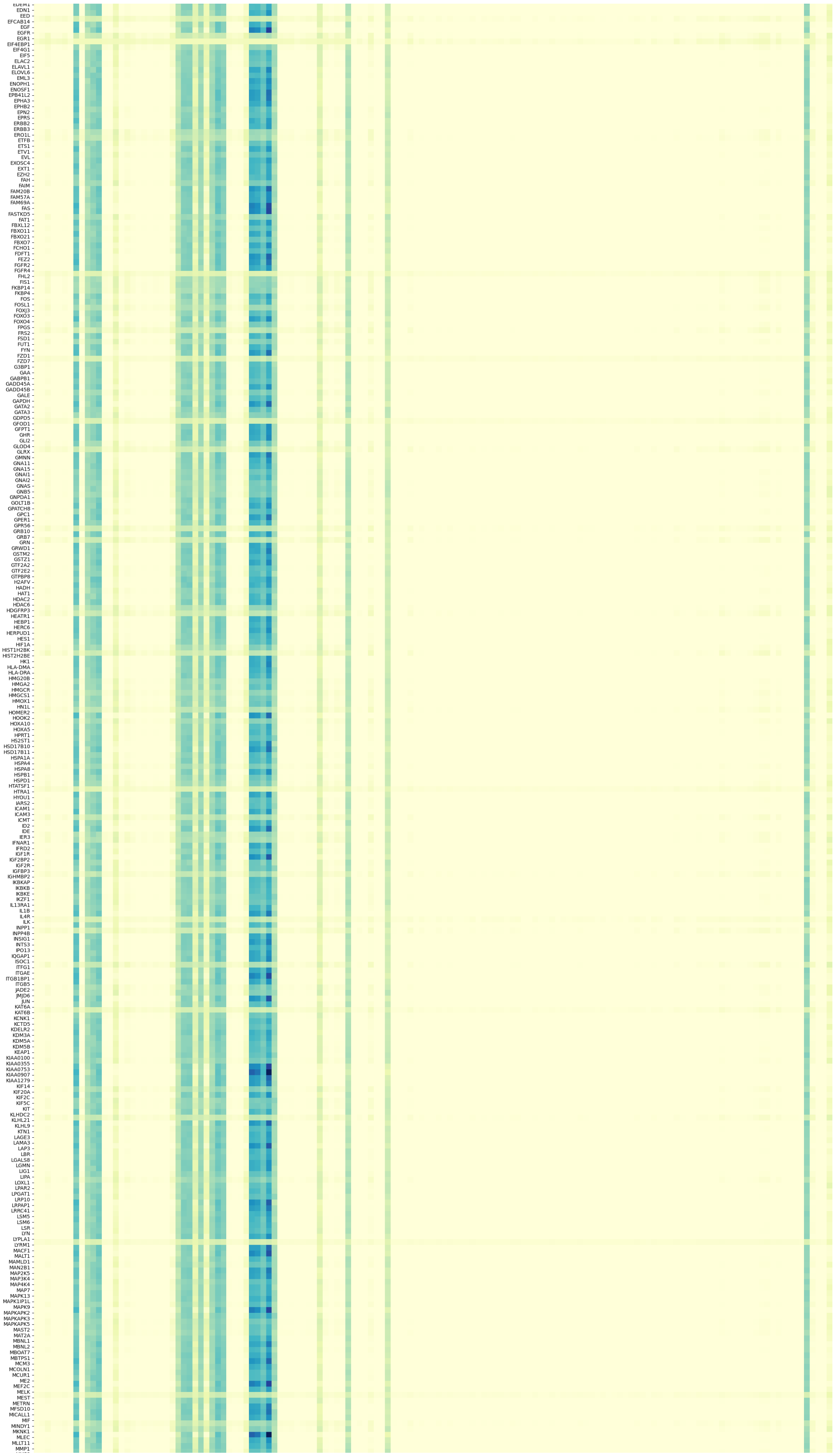

Fig. S6. Attention weights computed between the 978 shareable landmark gene embeddings and 140 perturbation-contextualized pathway embeddings, extracted from BADGER's Pathway-Gene Cross Attention Block when the input YAPC cell is treated with Atorvastatin (2/4 figure).

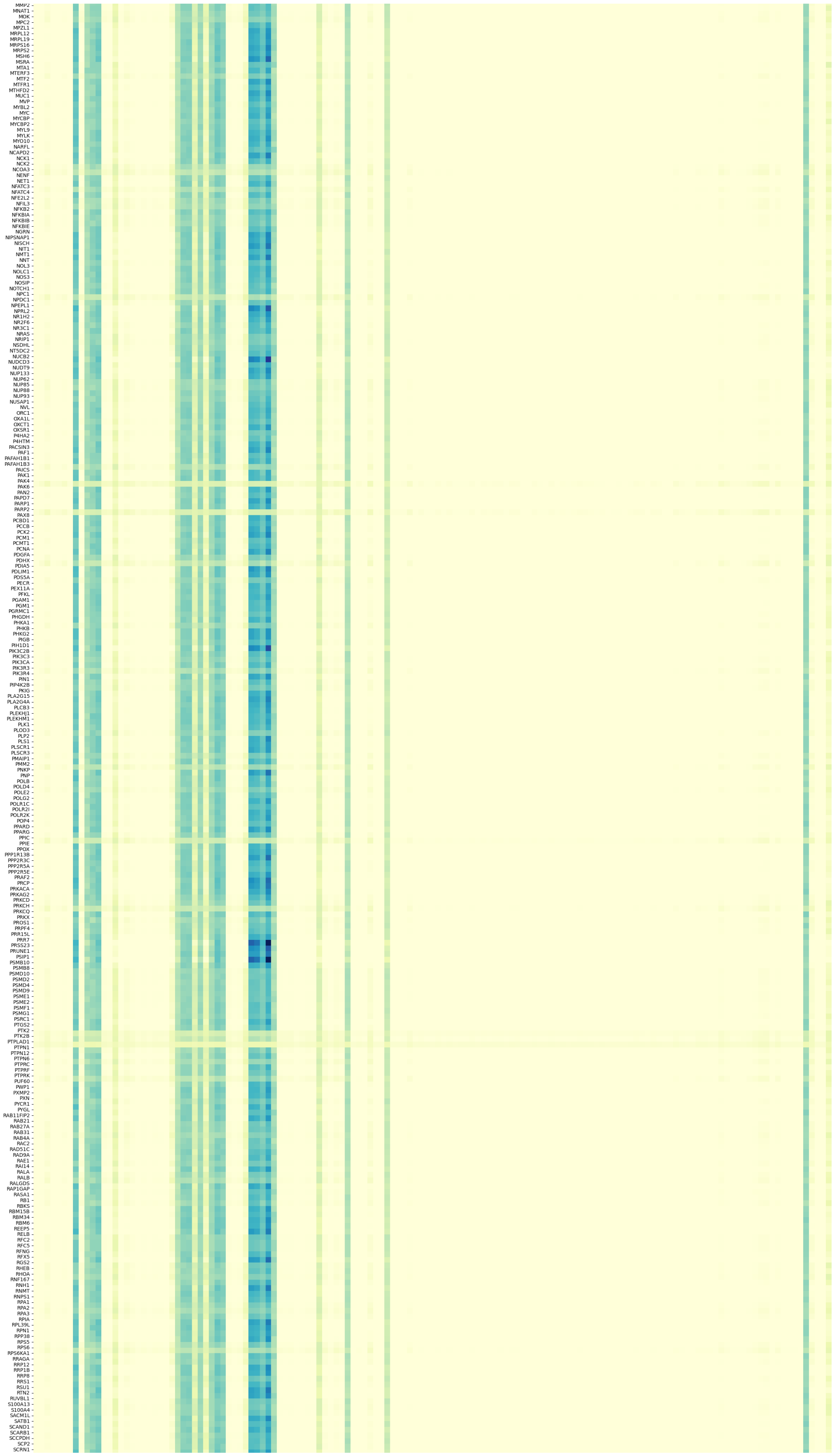

Fig. S7. Attention weights computed between the 978 shareable landmark gene embeddings and 140 perturbation-contextualized pathway embeddings, extracted from BADGER's Pathway-Gene Cross Attention Block when the input YAPC cell is treated with Atorvastatin (3/4 figure).

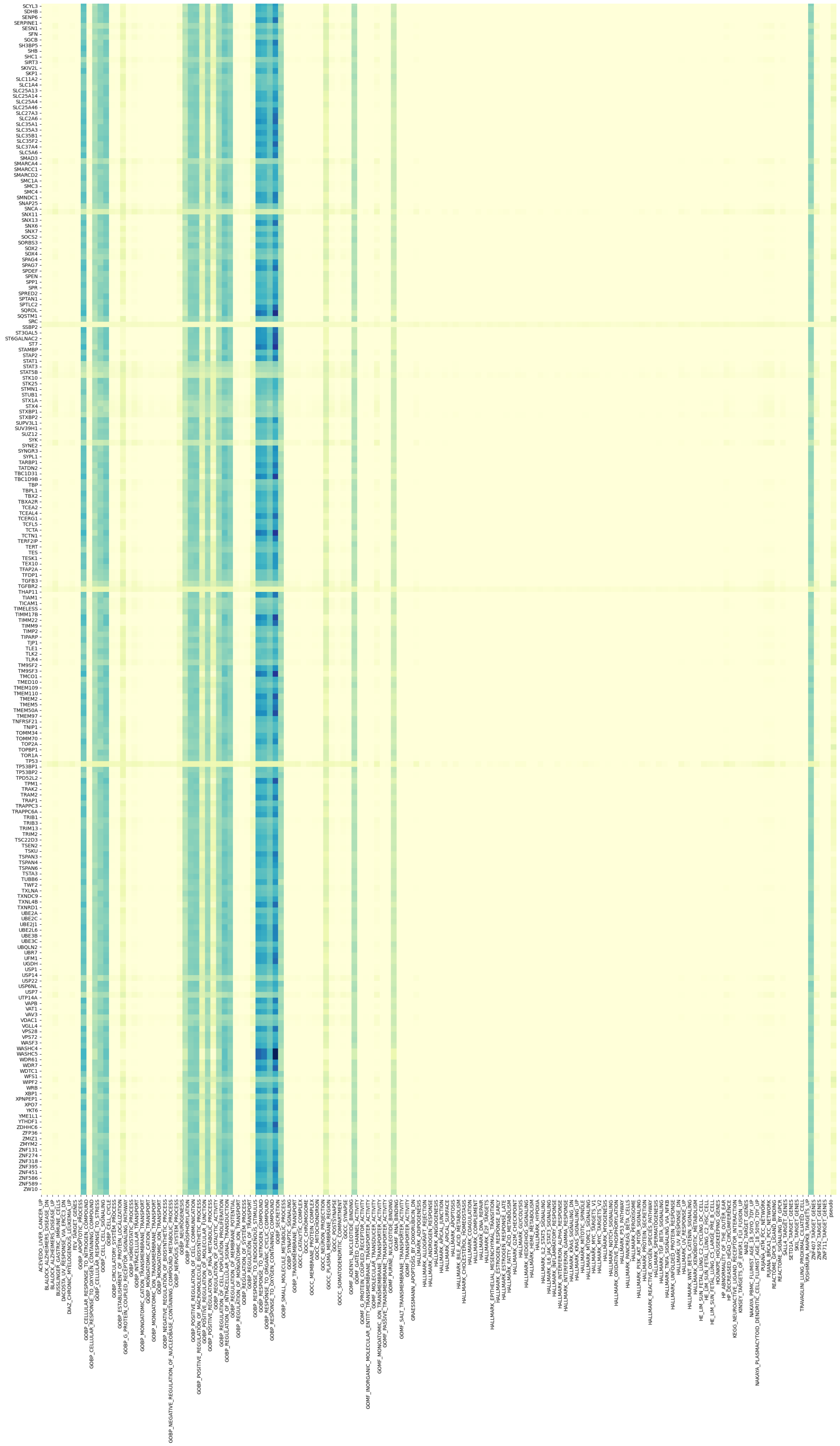

Fig. S8. Attention weights computed between the 978 shareable landmark gene embeddings and 140 perturbation-contextualized pathway embeddings, extracted from BADGER's Pathway-Gene Cross Attention Block when the input YAPC cell is treated with Atorvastatin (4/4 figure).

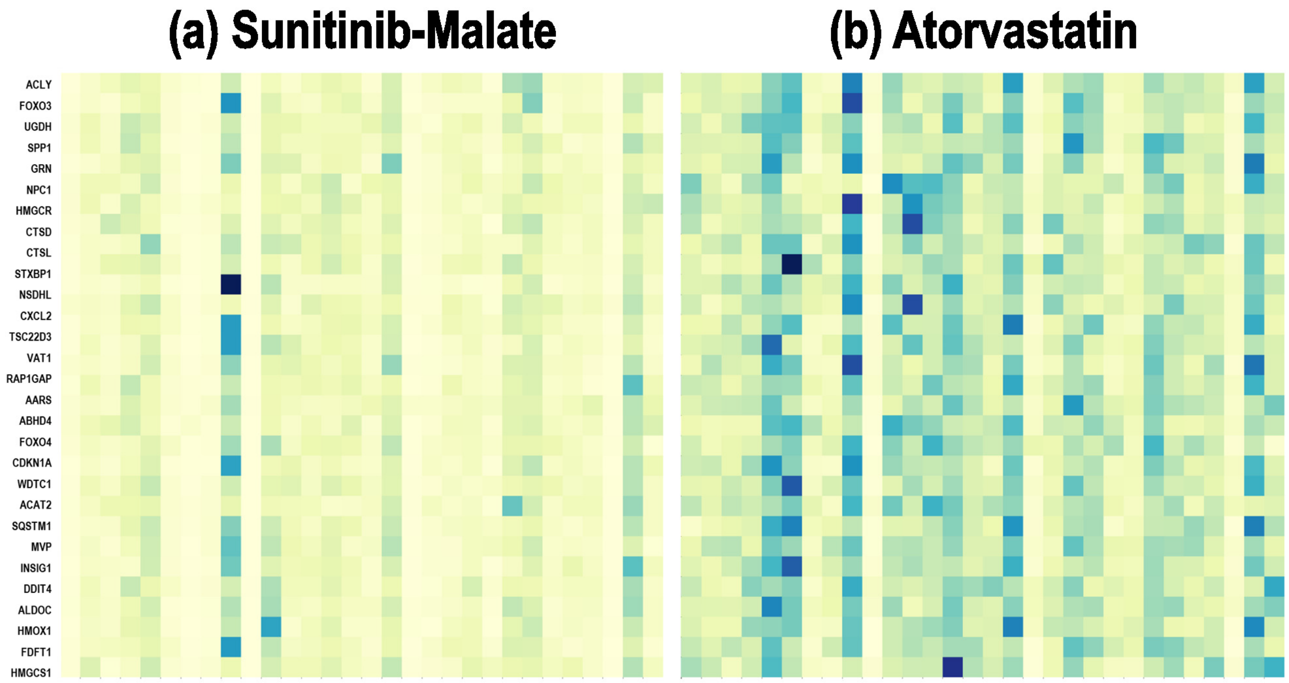

Fig. S9. Self-attention weights computed between the updated landmark gene embeddings where the genes were selected based on the intersection of the top 50 ranked predictions of up-regulated gene expressions made by BADGER when YAPC cells were treated with (a) Sunitinib and (b) Atorvastatin, respectively. Note that the DGE-based similarity score between the two drugs is 0.5810 which is shown in Table ??

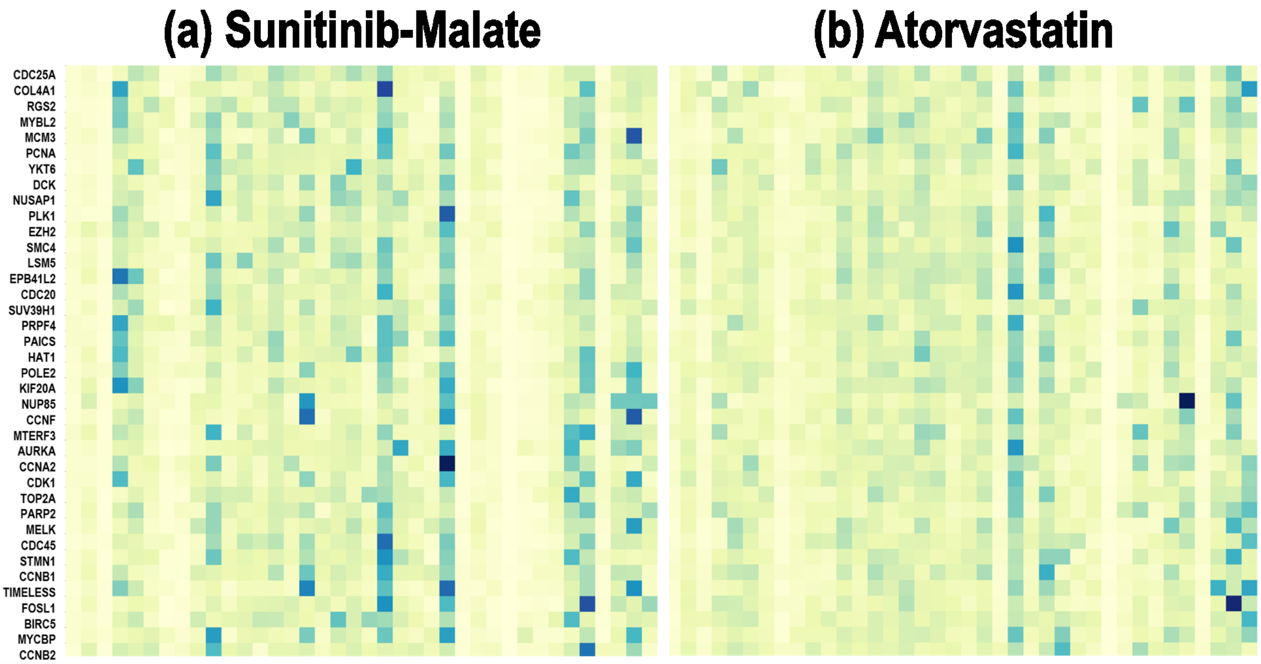

Fig. S10. Self-attention weights computed between the updated landmark gene embeddings where the genes were selected based on the intersection of the top 50 ranked predictions of down-regulated gene expressions made by BADGER when YAPC cells were treated with (a) Sunitinib and (b) Atorvastatin, respectively. Note that the DGE-based similarity score between the two drugs is 0.8884 which is shown in Table ??

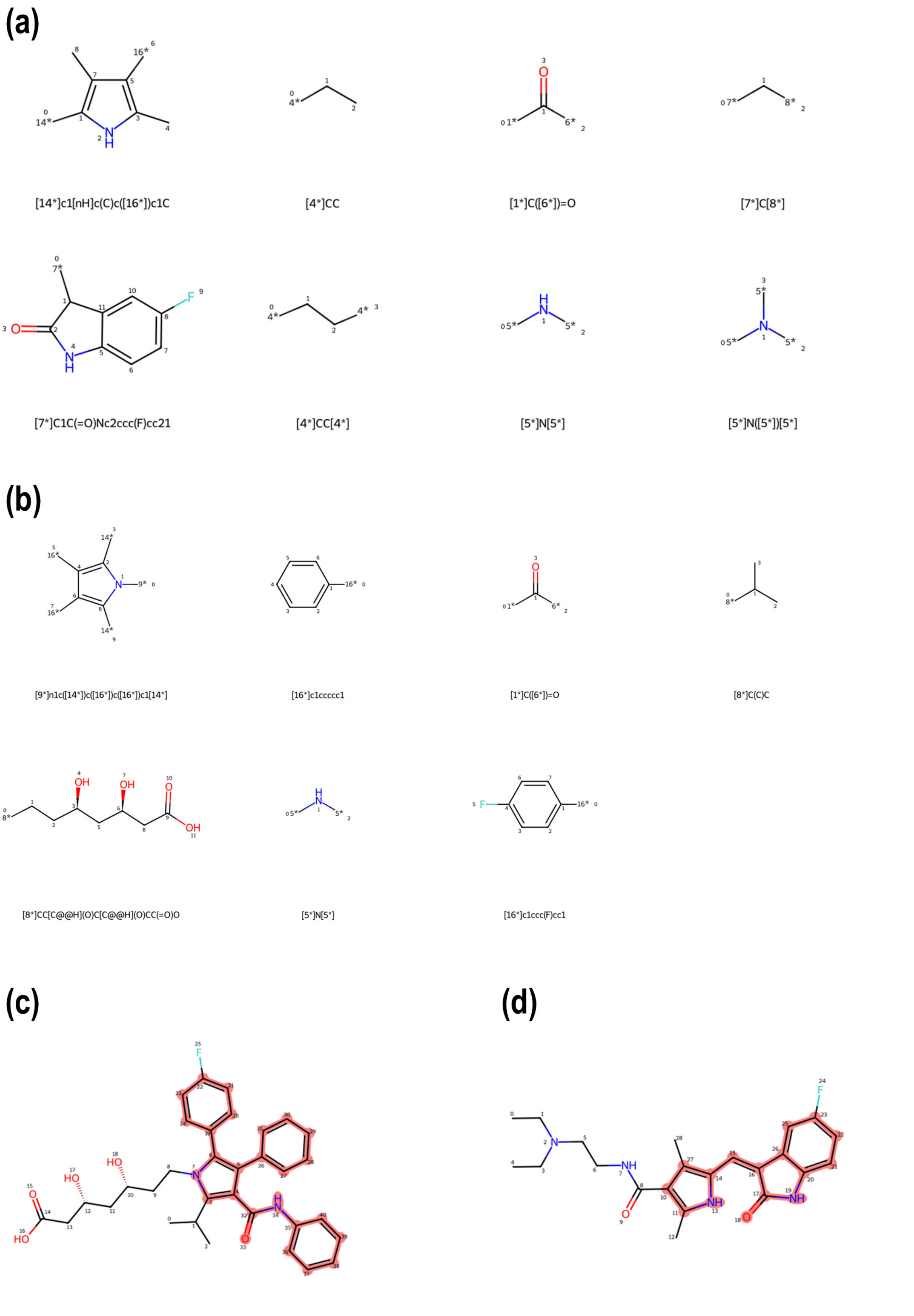

Fig. S11. Fragment analysis showing key structural components of Sunitinib Malate and Atorvastatin. (a) Molecular fragments of Sunitinib Malate. (b) Molecular fragments of Atorvastatin. (c) Highlighted fragments of Sunitinib-Malate - [14*]c1[nH]c(C)c([16*])c1C and [7*]C1C(=O)Nc2ccc(F)cc21 (d) Highlighted fragments of Atorvastatin - [9*]n1c([14*])c([16*])c([16*])c1[14*] and [8*]CCC@@HCC@@HCC(=O)O .

References

Barcelo, J., Samain, R., and Sanz-Moreno, V. (2023). Preclinical to clinical utility of rock inhibitors in cancer. *Trends in Cancer*.

Schweickert, P. G., Yang, Y., White, E. E., Cresswell, G. M., Elzey, B. D., Ratliff, T. L., Arumugam, P., Antoniak, S., Mackman, N., Flick, M. J., et al. (2021). Thrombin-par1 signaling in pancreatic cancer promotes an immunosuppressive microenvironment. *Journal of Thrombosis and Haemostasis*, 19(1):161–172.

Suo, L., Chang, X., Xu, N., and Ji, H. (2019). The anti-proliferative activity of gnrh through downregulation of the akt/erk pathways in pancreatic cancer. *Frontiers in endocrinology*, 10:370.
